# Investigation of Novel Benzoxazole-Oxadiazole Derivatives as Effective Anti-Alzheimer’s Agents: In Vitro and In Silico Approaches

**DOI:** 10.3390/ph16070909

**Published:** 2023-06-21

**Authors:** Saeed Anwar, Wajid Rehman, Rafaqat Hussain, Shoaib Khan, Mohammed M. Alanazi, Nawaf A. Alsaif, Yousaf Khan, Shahid Iqbal, Adeela Naz, Muhammad Ali Hashmi

**Affiliations:** 1 Department of Chemistry, Hazara University, Mansehra 21120, Pakistan; saeedanwar236u@gmail.com (S.A.); rafaqathussain0347@gmail.com (R.H.); 2 Department of Chemistry, Abbottabad University of Science and Technology (AUST), Abbottabad 22020, Pakistan; shoaibkhanswati@gmail.com; 3 Department of Pharmaceutical Chemistry, College of Pharmacy, King Saud University, Riyadh 11451, Saudi Arabia; mmalanazi@ksu.edu.sa (M.M.A.); nalsaif@ksu.edu.sa (N.A.A.); 4 Department of Chemistry, COMSATS University Islamabad, Islamabad 45550, Pakistan; yousaf7n@gmail.com; 5 School of Chemical and Environmental Engineering, College of Chemistry, Chemical Engineering and Materials Science, Soochow University, Suzhou 215123, China; shahidiqbal@suda.edu.cn; 6 Department of Chemistry, Division of Science & Technology, University of Education, Lahore 54770, Pakistan; adeelanaz943@gmail.com (A.N.); i4hashmi@hotmail.com (M.A.H.)

**Keywords:** benzoxazole, oxadiazole, anti-Alzheimer, in vitro and in silico molecular docking

## Abstract

Alzheimer’s disease (AD) is a progressive neurological illness that is distinguished clinically by cognitive and memory decline and adversely affects the people of old age. The treatments for this disease gained much attention and have prompted increased interest among researchers in this field. As a springboard to explore new anti-Alzheimer’s chemical prototypes, the present study was carried out for the synthesis of benzoxazole-oxadiazole analogues as effective Alzheimer’s inhibitors. In this research work, we have focused our efforts to synthesize a series of benzoxazole-oxadiazole (**1**–**19**) and evaluating their anti-Alzheimer properties. In addition, the precise structures of synthesized derivatives were confirmed with the help of various spectroscopic techniques including ^1^H-NMR, ^13^C-NMR and HREI-MS. To find the anti-Alzheimer potentials of the synthesized compounds (**1**–**19**), in vitro acetylcholinesterase (AChE) and butyrylcholinesterase (BuChE), inhibitory activities were performed using Donepezil as the reference standard. From structure-activity (SAR) analysis, it was confirmed that any variation found in inhibitory activities of both acetylcholinesterase (AChE) and butyrylcholinesterase (BuChE) enzymes were due to different substitution patterns of substituent(s) at the variable position of both acetophenone aryl and oxadiazole aryl rings. The results of the anti-Alzheimer assay were very encouraging and showed moderate to good inhibitory potentials with IC_50_ values ranging from 5.80 ± 2.18 to 40.80 ± 5.90 µM (against AChE) and 7.20 ± 2.30 to 42.60 ± 6.10 µM (against BuChE) as compared to standard Donepezil drug (IC_50_ = 33.65 ± 3.50 µM (for AChE) and 35.80 ± 4.60 µM (for BuChE), respectively. Specifically, analogues **2**, **15** and **16** were identified to be significantly active, even found to be more potent than standard inhibitors with IC_50_ values of 6.40 ± 1.10, 5.80 ± 2.18 and 6.90 ± 1.20 (against AChE) and 7.50 ± 1.20, 7.20 ± 2.30 and 7.60 ± 2.10 (against BuChE). The results obtained were compared to standard drugs. These findings reveal that benzoxazole-oxadiazole analogues act as AChE and BuChE inhibitors to develop novel therapeutics for treating Alzheimer’s disease and can act as lead molecules in drug discovery as potential anti-Alzheimer agents.

## 1. Introduction

**Figure 1:**
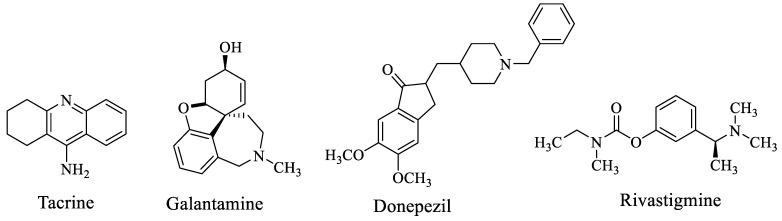
Available drugs of Alzheimer’s disease.

People in old age are the most affected by Alzheimer’s disease (AD), the most common kind of dementia and a fatal neurodegenerative condition marked by cognitive and memory issues [1,2,3]. It is a neurological condition that harms brain neurons and gradually reduces cognitive skills such as judgment, speech, memory and focus [4]. Although the mechanism of these symptoms is yet unknown, there is presently no conclusive therapeutic treatment for the progressive and cognitive deterioration caused by aging in AD [5]. In the world, there are 35 million people who suffer from AD, according to estimates. This number is anticipated to increase to 65 million by 2030 and to 115 million by 2050 [6,7]. These figures emphasize how critical it is to create a successful treatment. The most important pathogenic features of AD are the loss of cholinergic neurons in the basal frontal cortex; intracellular neurofibrillary tangles brought on by oxidative stressors, τ-protein hyperphosphorylation and extracellular β-amyloid (βA) plaques [8,9]. One factor that is given a substantial role in AD is inadequate cholinergic transmission, which results in the formation of behavioral, functional and cognitive symptoms. According to the cholinergic theory, this insufficient quantity of acetylcholine in the brain will cause cholinesterase enzymes to be inhibited [10,11,12]. As a result, using receptor agonists or cholinesterase (ChE) inhibitors, the goal of therapy is frequently to improve cholinergic system dysfunction [13]. Rivastigmine, galantamine and Donepezil are FDA-approved drugs based on the “one drug–one target” approach that were currently used to treat Alzheimer candidates by blocking AChE, underlining the shortcomings of this approach for the complex nature of AD. Whereas, the most advanced therapeutic technique now in use are based on the “one drug–many targets” strategy which encourages the use of medications having numerous effects at various target locations [14,15]. Donepezil is the drug that is most frequently prescribed and most efficient for treating all stages of AD. AChE is effectively suppressed as a result of its binding to both the catalytic and peripheral anionic sites. This “double binding” feature should be considered while creating new drugs and treatment plans. In this study, taking into account the chemical makeup of the Donepezil molecule, we created, synthesized and examined the biological effects of novel compounds comprising piperazine and different secondary amine derivatives. We also analyzed relevant studies on molecular modeling [14,15]. Several drugs have been approved by the food and drug administration (FDA) for patients with Alzheimer disease (Figure 1) including galantamine and Donepezil which are selective for AChE while rivastigmine and tacrine were found to inhibit both BuChE and AChE [16].

**Figure 2:**
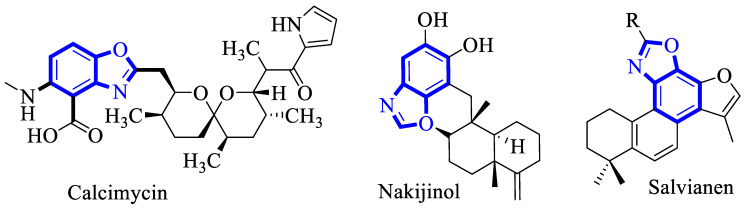
Structure of some biologically active benzoxazole alkaloids.

Benzoxazole has been reported as a privileged scaffold and is widely distributed in cutting-edge materials, natural products, biologically active and pharmaceuticals drugs [16,17,18]. For instance, the benzoxazole alkaloids that are derived from marine sponges or plants have known as potent therapeutic agents (Figure 2) [19,20,21,22,23].

**Figure 3:**
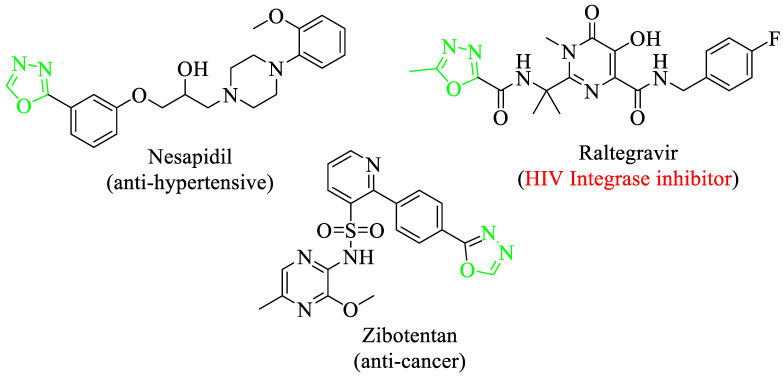
Chemical structure of biological active drugs of oxadiazole.

1,3,4-Oxadiazole derivatives were known to have a diverse range of applications not only for drug discovery purposes but also showed numerous pharmacological and biological activities [24,25] including herbicidal [26], antibacterial [27], insecticidal [28] and anti-inflammatory [29] profiles. In addition, a 1,3,4-oxadiazole core structure containing scaffolds also finds applications as anti-bacterial agents against a variety of bacterial pathogens (plants) such as *Xac* [30], *Xoo* [31] and *R. solanacearum* [32]. Moreover, there are numerous biologically potent drugs that contain oxadiazole skeleton in their core structure like Zibotentan (anti-cancer agent), Railtegraviras (HIV Integrase inhibitor) and nesapidil (antihypertensive) (Figure 3) [33].

**Figure 4:**
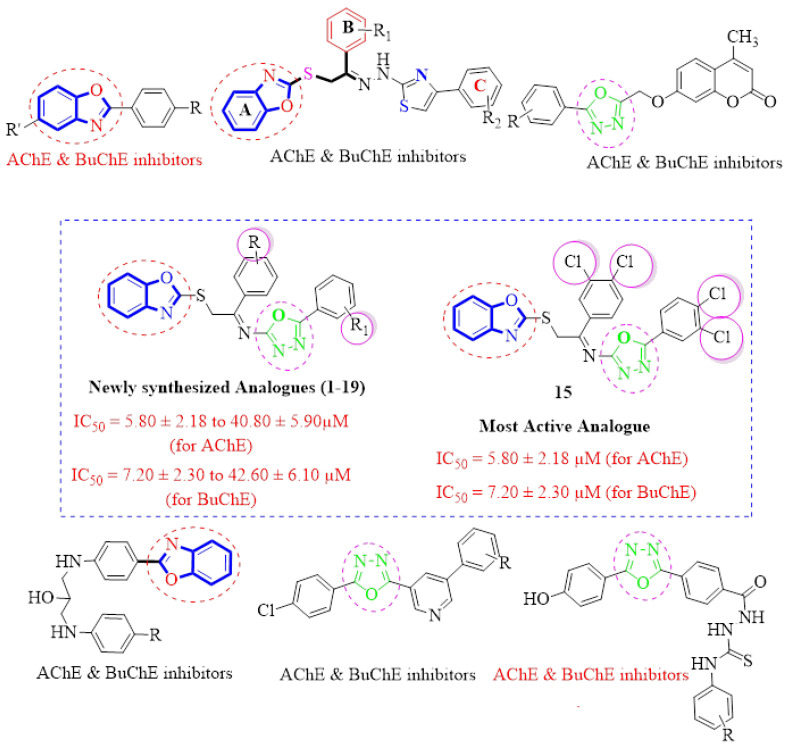
Design strategy of the target molecules [35,36,37,38,39,40].

The molecular hybridization approach has been widely employed for the design and synthesis of hybrid analogues for the treatment of Alzheimer’s disease. This approach mainly involves the combining of two or more different pharmacophore moieties in a single molecule having a common skeleton. These hybrid molecules may have advantages over standard drugs [34]. In our current work, we are using molecular hybridization to combine the biologically important two heterocyclic moieties; benzoxazole and oxadiazole, to get new hybrid molecules (Figure 4). As earlier discussed, both benzoxazole and oxadiazole moieties are very important for the treatment of Alzheimer’s disease and thus hybrid analogues containing benzoxazole and oxadiazole moieties were synthesized and evaluated for their in vitro acetylcholinesterase (AChE) and butyrylcholinesterase (BuChE) and molecular docking studies thereafter.

## 2. Results and Discussion

### 2.1. Chemistry

**Scheme 1:**
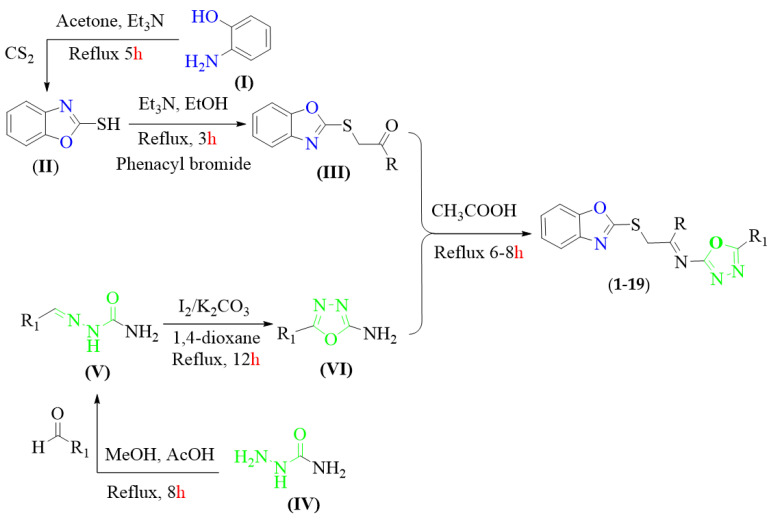
Synthesis of benzoxazole-based oxadiazole hybrids scaffolds (**1**–**19**).

As shown in Scheme 1, the target compounds were afforded via five steps. First of all, 2-aminophenol (I) was treated with carbon disulfide in acetone along with a catalytic amount of triethylamine. The reaction mixture was refluxed and stirred for 5 h to obtain the 2-marcaptobenzoxazole (II) substrate which was further treated with different substituted phenacyl bromide in ethanol and triethylamine. After being stirred for 3 h, the solvent was evaporated under reduced pressure to afford S-substituted benzoxazole intermediate (III).

Alternatively, semicarbazide compound (IV) and appropriate benzaldehyde derivatives in methanol and acetic acid were refluxed for 8 h over a pre-heated sand bath and precipitate product were filtered off as semicarbazone intermediate (V) which was further undergoing an oxidative cyclization in 1,4-dioxane with iodine and potassium carbonate. The reaction residue was stirred until the formation of 2-amino-oxadiazole (VI) was complete (TLC was employed to check the progress of the reaction and reflux 12 h). 

Encouraged by the success of S-substituted benzoxazole (III) and 2-amino-oxadiazole (VI) intermediates, we sought to further extend the scope and diversity of this practical approach by treating S-substituted benzoxazole (III) with 2-amino-oxadiazole (VI) in acetic acid and refluxed the reaction mixture for 6–8 h. Upon completion, the solvent was evaporated under reduced pressure to obtain targeted benzoxazole-bearing oxadiazole hybrids analogues (**1**–**19**) with the appropriate yield. The precise structures of all the synthesized scaffolds were confirmed using various spectroscopic tools including HREI-MS and NMR (^1^H- and ^13^C-NMR) (Scheme 1).

The proton NMR spectrum of compound **17** was recorded in DMSO-*d*_6_ on a Bruker operating at 600 MHz instrument. The two doubles of doublet (dd) were observed for different benzoxazole protons present at the 4- and 7-positions of the ring with chemical shift values resonating at 8.47 (dd, *J* = 7.0, 2.3 Hz) and 8.39 (dd, *J* = 7.9, 2.0 Hz), respectively. Whereas, the remaining two protons of benzoxazole present at the 5- and 6-positions of the ring coupled to their neighboring ortho-protons and give two distinct triplets appearing at chemical shift values of 8.08 (t, *J* = 7.5 Hz) and 7.94 (t, *J* = 7.4 Hz), correspondingly. Furthermore, a singlet was also observed for two active methylene protons –CH_2_- attached between the sulfur and aromatic acetophenone aryl ring at δ_H_ 3.62 (s, 2H, -S-CH_2_). The molecule comprises two aromatic rings termed as acetophenone aryl (R) and oxadiazole aryl (R_1_) rings. Among oxadiazole aryl ring protons, doublet was appeared at δ_H_ 8.61 for two protons present at ortho- to oxadiazole ring, while the other two protons which are meta- to the oxadiazole ring were resonated at δ_H_ 8.22 (d, *J* = 8.0 Hz, 2H, Ar-H) as doublets, respectively. On the other hand, a aromatic proton present at 2-position of acetophenone aryl ring resonated at δ_H_ 8.81 (s, 1H, Ar-H) as singlet. Besides that, two different doublets (d) were observed for remaining two acetophenone aryl protons present at the 5- and 6-positions of the ring with chemical shift values resonating at δ_H_ 8.78 and 8.72, respectively.

### 2.2. In Vitro Acetylcholinesterase (AChE) and Butyrylcholinesterase (BuChE) Inhibitory Activities

Benzoxazole-based oxadiazole analogues (**1**–**19**) were subjected to in vitro acetylcholinesterase (AChE) and butyrylcholinesterase (BuChE) activities for the first time. It is interesting to reveal that the entire series of benzoxazole-oxadiazole were found to be active with IC_50_ values ranging between 5.80 ± 2.18 to 40.80 ± 5.90 µM (against AChE) and 7.20 ± 2.30 to 42.60 ± 6.10 µM (against BuChE) as compared to standard Donepezil drug (IC_50_ = 33.65 ± 3.50 µM (for AChE) and 35.80 ± 4.60 µM (for BuChE), respectively. Specifically, analogues **2**, **15** and **16** were recognized as considerably active, even identified to be more active than standard Donepezil inhibitors with IC_50_ values of 6.40 ± 1.10, 5.80 ± 2.18 and 6.90 ± 1.20 (against AChE) and 7.50 ± 1.20, 7.20 ± 2.30 and 7.60 ± 2.10 (against BuChE). However, the rest of the synthesized benzoxazole-oxadiazole analogues also displayed good to moderate inhibitory potentials against targeted enzymes.

#### Structure-Activity Relationship (SAR) Studies for Acetylcholinesterase (AChE) and Butyrylcholinesterase (BuChE) Inhibitory Activities

For the sake of SAR simplification, it was discussed based on the variation of position, number/s and nature of attached substituent(s) (R and R_1_) around aryl rings.

**Figure 5:**
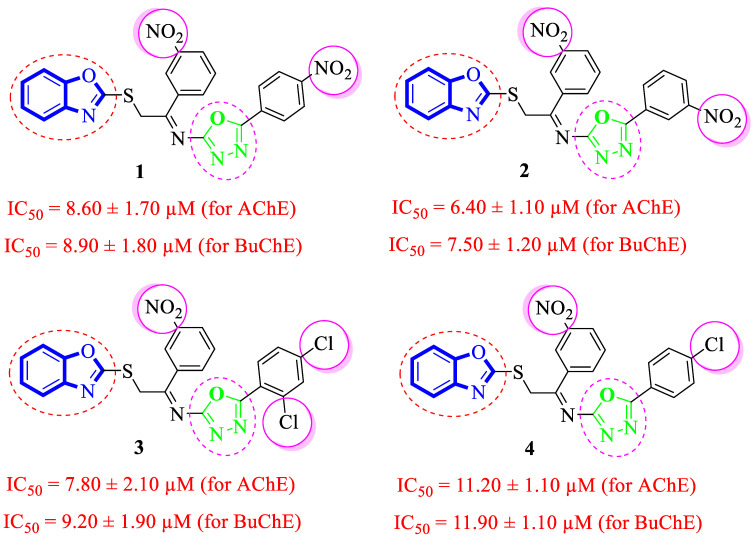
Comparative study of nitro-substituted analogues **1**–**4**.

It was noteworthy that analogues **1**–**4** were found to be encouraging for the inhibition of acetylcholinesterase (AChE) and butyrylcholinesterase (BuChE) enzymes. Among them, analogue **2** (bearing –NO_2_ moiety on *meta*-position of both aryl rings) was found to be significantly active, even more potent than the standard Donepezil drug. Moving the –NO_2_ group from the 3-position of the oxadiazole aryl ring to its 4-position as in analogue **1** (bearing 3-NO_2_ on acetophenone-derived aryl ring and 4-NO_2_ group on the oxadiazole aryl ring) showed a slightly decreased inhibitory activities against both targeted AChE and BuChE enzymes. Analogues **3** and **4** bearing both –NO_2_ and –Cl substitution demonstrated decreased AChE and BuChE inhibitory activities relative to –NO_2_ substituted analogues **1** and **2**. It is interesting to mention that analogue **3** (that holds the 3-NO_2_ on acetophenone-derived aryl ring and 2,4-diCl substitution on the oxadiazole aryl ring) is found to be more active than analogue **4** (bearing 4-Cl moiety on the oxadiazole aryl ring along with the 3-NO_2_ group on another aryl ring) which demonstrated that the mono-Cl group does not really work like di-Cl groups in acetylcholinesterase (AChE) and butyrylcholinesterase (BuChE) inhibition. Further, a comparison of analogue **1** (that had a 4-NO_2_ group on the oxadiazole aryl ring) with analogue **4** (bearing 4-Cl group on oxadiazole aryl ring) demonstrated that analogue **1** showed more inhibitory potential than analogue **4** against targeted enzymes. This showed that the alteration in the nature of the attached substituent on the oxadiazole aryl ring which may cause alteration in inhibitory potentials against targeted acetylcholinesterase and butyrylcholinesterase enzymes (Figure 5).

**Figure 6:**
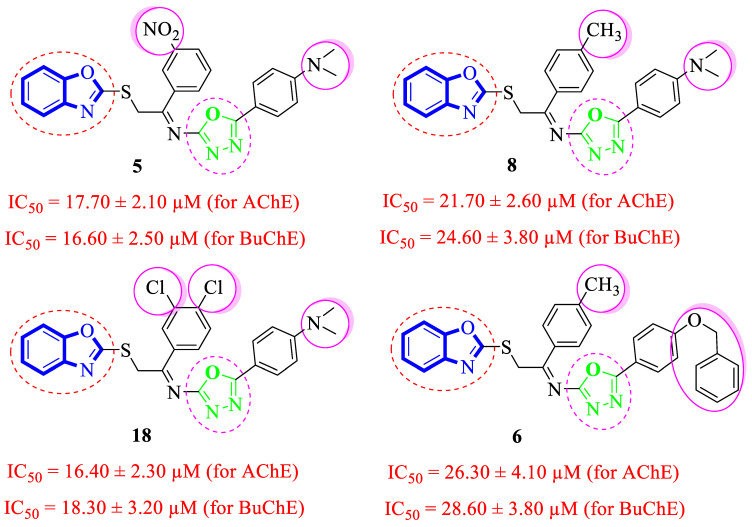
Comparative study of di-methylamino and phenyloxy-substituted analogues **5**, **6**, **8** and **18**.

Analogue **8** bearing 4-CH_3_ moiety on one aryl ring and the 4-dimethylamino group at the oxadiazole aryl ring emerged as more active than the standard Donepezil inhibitor. The replacement of the 4-dimethylamino group of analogue **8** with the 4-benzyloxy group leads to a decline in the acetylcholinesterase and butyrylcholinesterase inhibitory potential of analogue **6**. However, the comparison of analogue **8** (having 4-CH_3_ moiety on the acetophenone-derived aryl ring) with analogue **5** (with 3-NO_2_ group on acetophenone-derived aryl ring) demonstrated that analogue **5** emerged as a superior inhibitor of the acetylcholinesterase and butyrylcholinesterase enzymes when compared to analogue **8**, which shows that the methyl group bearing analogue **8** is not able to attain the conformations to fit well in the active sites of targeted enzymes. Moreover, the acetylcholinesterase and butyrylcholinesterase activities were further enhanced by changing the 3-NO_2_ group of analogue **5** with the 3,4-diCl moieties as in analogue **18**, i.e., the addition of the 3,4-diCl moieties as in analogue **18** leads to slightly improved acetylcholinesterase and butyrylcholinesterase inhibitory potential (Figure 6).

**Figure 7:**
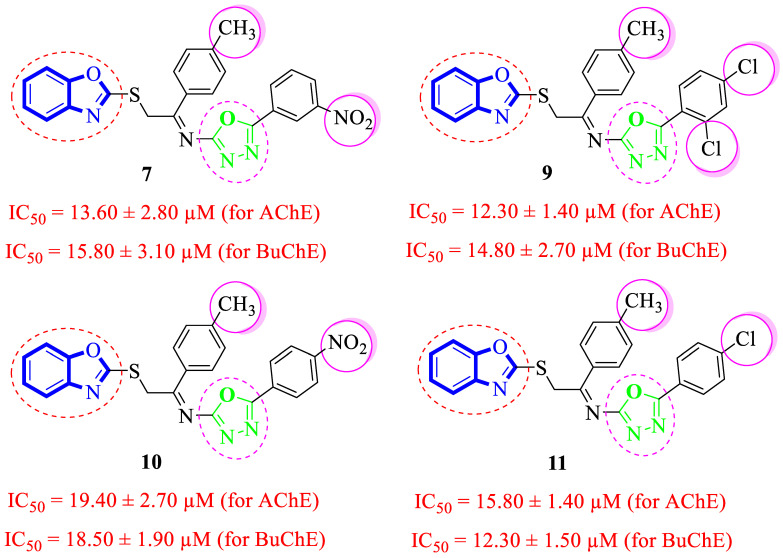
Comparative study of methyl-substituted analogues **7**, **9**, **10** and **11**.

Analogues **9**–**11** and **7** possessing –CH_3_ substituent at the 4-position of acetophenone-derived aryl ring and a variety of other groups such as –Cl and –NO_2_ groups at the oxadiazole aryl ring demonstrated considerable inhibition for acetylcholinesterase and butyrylcholinesterase enzymes. Among these analogues, the analogue **9** bearing 2,4-diCl substitutions at the oxadiazole aryl ring showed excellent acetylcholinesterase and butyrylcholinesterase inhibitory activities. The de-attachment of the 2-Cl substituent as in analogue **11** leads to slightly decreased acetylcholinesterase and butyrylcholinesterase inhibitory activities. 2,4-diCl substituted into analogue **9** demonstrated almost similar inhibitory activity to *meta*-NO_2_ substituted analogue **7**. However, the nitro-substituted positional isomer of analogue **7** (that holds the 3-NO_2_ moiety at oxadiazole aryl ring), that is, **10** (bearing 4-NO_2_ substitution at oxadiazole aryl ring) showed less inhibitory potentials, which demonstrate that –NO_2_ substitution at the 4-position does not really work like –NO_2_ moiety at the 3-position in acetylcholinesterase and butyrylcholinesterase inhibition (Figure 7).

**Figure 8:**
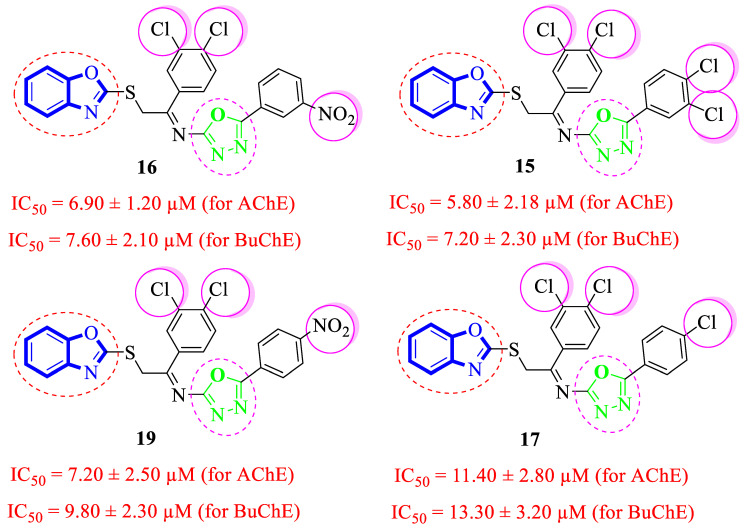
Comparative study of di-Cl substituted analogues **15**–**17** and **19**.

Analogue **15** bearing 3,4-diCl substitutions at both aryl rings was identified as the most active compound of the series. Further, the comparison of 3,4-diCl substituted into analogue **15** with analogue **17** (that holds the 4-Cl moiety at the oxadiazole aryl ring) demonstrated that the dichloro-substituted analogue **15** showed better acetylcholinesterase and butyrylcholinesterase inhibitory potential relative to the monochloro-substituted analogue **17**. However, the analogue **16** (having a *meta*-NO_2_ substitution at oxadiazole aryl ring along with the 3,4-dichloro substitutions at the acetophenone-derived aryl ring) emerged as the second most active analogue, even though many folds are more potent than standard Donepezil drug. Nonetheless, the positional isomer **19** (bearing 4-NO_2_ moiety at oxadiazole aryl ring) showed less inhibitory potential relative to analogue **16**. This activity comparison confirmed that –NO_2_ substitution at the 3-position of the oxadiazole aryl ring uplifts the inhibitory strength to a good extent against targeted AChE and BuChE enzymes (Figure 8).

The structure–activity relationship (SAR) showed that electron withdrawing groups like –NO_2_ and –Cl moieties play an important role in significant acetylcholinesterase and butyrylcholinesterase activities of the synthesized compounds. However, the other groups also demonstrated better activities when placed in certain positions and numbers.

### 2.3. Molecular Docking Study

**Figure 9:**
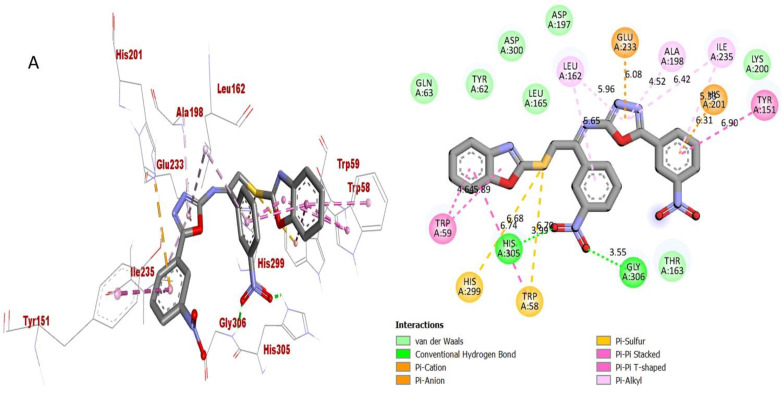
Protein–ligand interaction (PLI) profile of the second most active analogue; (**A**) for analogue-**2** against acetylcholinesterase enzyme and its 3D (**left**) and 2D (**right**) diagram.

**Figure 10:**
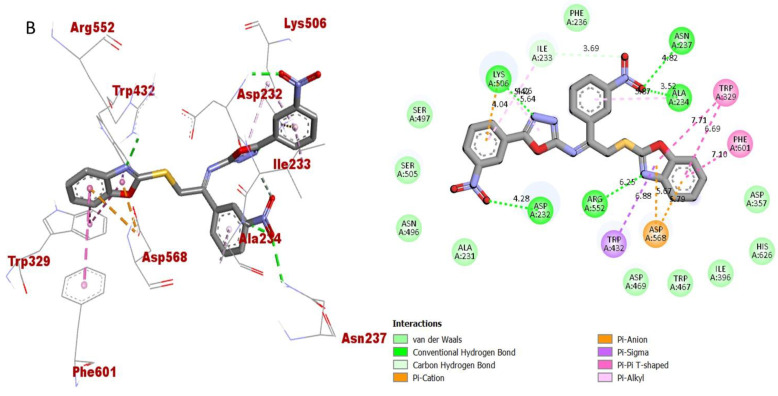
Protein-ligand interaction (PLI) profile of 2nd most active analogue; (**B**) for analogue-**2** against butyrylcholinesterase enzyme and its 3D (**left**) and 2D (**right**) diagram.

**Figure 11:**
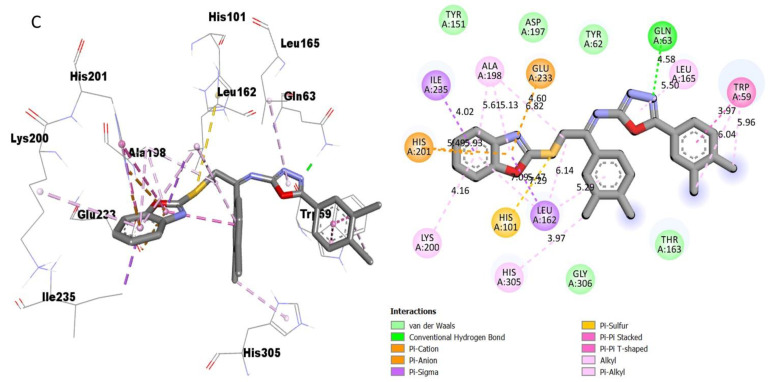
Protein–ligand interaction (PLI) profile of the most active analogue; (**C**) for analogue-**15** against acetylcholinesterase enzyme and its 3D (**left**) and 2D (**right**) diagram.

**Figure 12:**
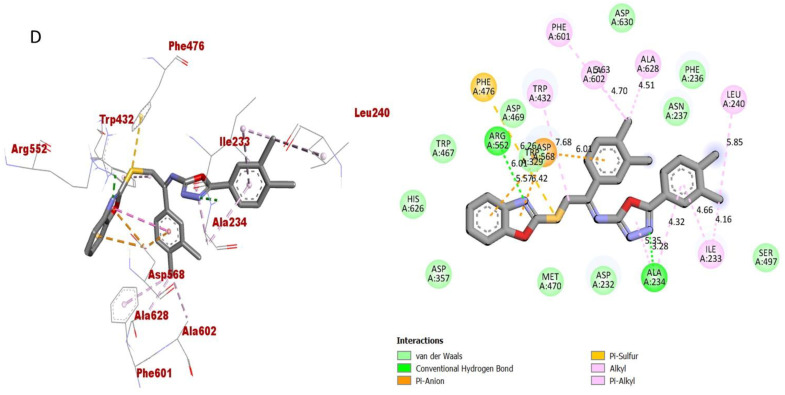
Protein–ligand interaction (PLI) profile of the most active analogue; (**D**) for analogue-**15** against butyrylcholinesterase enzyme and its 3D (**left**) and 2D (**right**) diagram.

**Figure 13:**
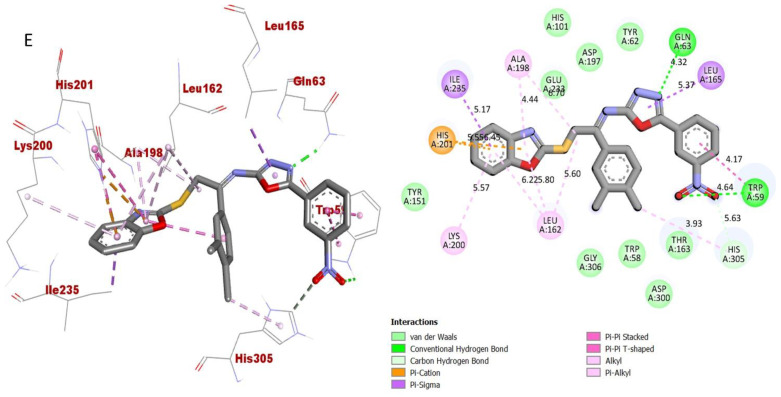
Protein–ligand interaction (PLI) profile of the third most active analogue; (**E**) for analogue-**16** against acetylcholinesterase enzyme and its 3D (**left**) and 2D (**right**) diagram.

**Figure 14:**
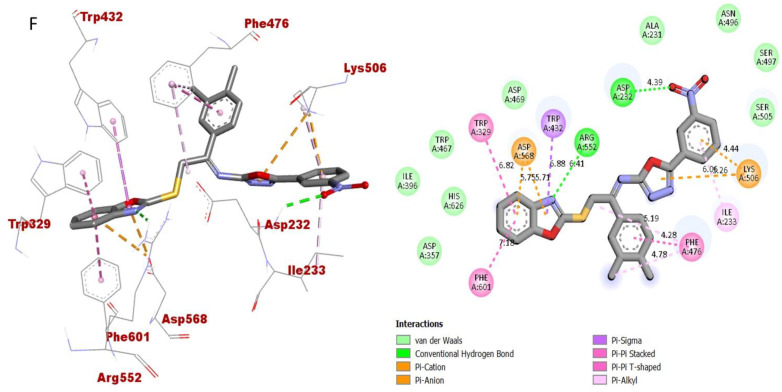
Protein–ligand interaction (PLI) profile of the third most active analogue; (**F**) for analogue-**16** against butyrylcholinesterase enzyme and its 3D (**left**) and 2D (**right**) diagram.

In order to correlate well between the in vitro experiment and the in silico computational results, a molecular docking study was performed on most active analogues **2**, **15** and **16** against targeted acetylcholinesterase and butyrylcholinesterase enzymes and the results obtained demonstrated that these active analogues furnished several key interactions with the active sites of targeted enzymes and hence enhanced the enzymatic activities. Further, the key interactions established by the most active analogues with the catalytic cavity of the targeted acetylcholinesterase and butyrylcholinesterase enzymes are shown in (Table 1, Figure 9, Figure 10, Figure 11, Figure 12, Figure 13 and Figure 14).

The synthesized scaffolds and their inhibitory profile against the AChE and BuChE enzymes are listed in (Table 2). It was found that the position, type and number of functional moieties connected to the aryl portion of the benzoxazole-oxadiazole skeleton were substantially correlated with the IC_50_ values of AChE and BuChE inhibitors. Molecular docking was used to explore the position, kind and quantity of the attached substituents as well as enzymatic inhibition, as well as to further develop the binding contacts of the newly provided scaffolds with active residues of both targeted AChE and BuChE enzymes. The detailed PLI analysis of the most potent analogues such as **2**, **15** and **16** against AChE and BuChE enzymes revealed that they had established a number of significant interactions with the active residues of targeted AChE and BuChE enzymes. These interactions may have improved the inhibitory profile of these active analogues against the targeted AChE and BuChE. The second most active analogue **2** established several key interactions with catalytic residues of AChE enzyme including TRP-A-58, HIS-A-305, HIS-A-299, TRP-A-59, TRP-A-59, LEU-A-162, GLU-A-233, ALA-A-198, ILE-A-235, HIS-A-201, TYR-A-151 and GLY-A-306 (Figure 9), while against BuChE, this analogue 2 also adopted numerous important interactions including ASP-A-232, LYS-A-506, ILE-A-233, ASN-A-237, ALA-A-234, TRP-A-329, PHE-A-601, ASP-A-568, TRP-A-432 and ARG-A-552 (Figure 10).

However, analogue **15** (bearing di-Cl substitutions at the 3,4-position of both aryl rings) was identified as the most potent inhibitor of targeted AChE and BuChE enzymes among the current synthesized series. The detailed protein–ligand interaction (PLI) profile of the most active analogue **15** against the AChE enzyme showed a number of significant contacts with the enzyme’s active site including residues HIS-A-305, LEU-A-162, LEU-A-162, HIS-A-101, LYS-A-200, HIS-A-201, ILE-A-235, ALA-A-198, GLU-A-233, GLN-A-63, LEU-A-165 and TRP-A-59 (Figure 11), while against the BuChE, this compound **15** holds several key interactions such as ARG-A-552, PHE-A-476, ASP-A-568, ASP-A-568, TRP-A-432, PHE-A-601, ALA-A-602, ALA-A-628, LEU-A-240, ILE-A-233 and ALA-A-234 (Figure 12). Due to the attached electron-withdrawing groups (3,4-di-Cl groups) to both aryl parts, which largely remove the electronic density from the Ph-ring and make it more susceptible to interactions with the active site of the AChE and BuChE enzyme and also capable of side-wise association with catalytic residues of targeted enzymes.

As for the third most active analogue **16** (holds 3,4-diCl substitutions at one aryl ring and 3-NO_2_ group on another aryl ring), the detailed protein–ligand interaction profile (PLI) demonstrated numerous key interactions with the catalytic cavity of AChE enzymes such as LEU-A-162, LYS-A-200, HIS-A-201, ILE-A-235, ALA-A-198, GLN-A-63, LEU-A-165, TRP-A-59 and HIS-A-305 (Figure 13), while against the BuChE, this analogue also established important interactions including PHE-A-601, TRP-A-329, ASP-A-568, TRP-A-432, ARG-A-552, ASP-A-232, LYS-A-506, PHE-A-476, LYS-A-506, ILE-A-233 and PHE-A-476 (Figure 14).

### 2.4. Computational Details

For all the computations of compounds **1**, **2**, **15**–**17**, **19** and standard Donepezil, Gaussian 16 software was used [41]. For geometry optimizations, Ahlrich’s triple ζ zeta basis set def2TZVP with the Adamo’s hybrid version of Perdew, Burke and Ernzerh of functional (PBE0) was used [42]. Frequencies were calculated on these optimized structures at PBE0-D3BJ/def2TZVP which also confirmed that the optimized molecules are true minima. Grimme’s empirical D3 correction with Becke Johnson damping (D3BJ) was implemented on all the calculations [43,44]. The frequency calculations confirmed the optimized structures to be true minima on the potential energy surface. The 3D imaging of molecular orbital was conducted using Gauss View 6.0 and for molecular structure visualization, CYL view software was used. A frontier molecular orbital (FMOs) analysis and the parameters used to describe the reactivity of molecules were calculated at method same as frequency calculation. Nonlinear optical (NLO) properties along with polarizability parameters were evaluated using the same level of theory as described for the optimizations (Table 3).

#### 2.4.1. Frontier Molecular Orbital (FMO) Analysis

To learn more about the electronic properties and reactivity of compounds, an FMO analysis is carried out. The kinetic stability and reactivity of compounds are confirmed by the HOMO-LUMO gaps (Δ_H-L_), which are explained by the energies of HOMO (E_HOMO_) and LUMO (E_LUMO_) in addition to electronic characteristics. Large Δ_H-L_ indicates the low reactivity and greater stability of a molecule and vice versa [45]. HOMO energies (E_HOMO_), LUMO energies (E_LUMO_) and HOMO–LUMO energy gaps (Δ_H-L_) of all compounds are concluded in Table 4.

Among the compounds of **1**, **2**, **15**–**17**, **19** and the standard drug, **1** and **19** have the smallest Δ_H-L_, i.e., 3.86 and 3.88 eV, respectively. Hence, **1** and **19** are considered to be a little less stable and relatively reactive among all the synthesized compounds. For substituted benzoxazole, oxadiazole and benzoxazole-based compounds, HOMO-LUMO gaps have previously been observed ranging from 0.63 to 3.0 eV. They are said to be highly durable substances [46]. On the other hand, the standard drug has a Δ_H-L_ of 4.61 eV. This value represents greater stability and lesser reactivity as compared to **1** and **19** compounds.

**Figure 15:**
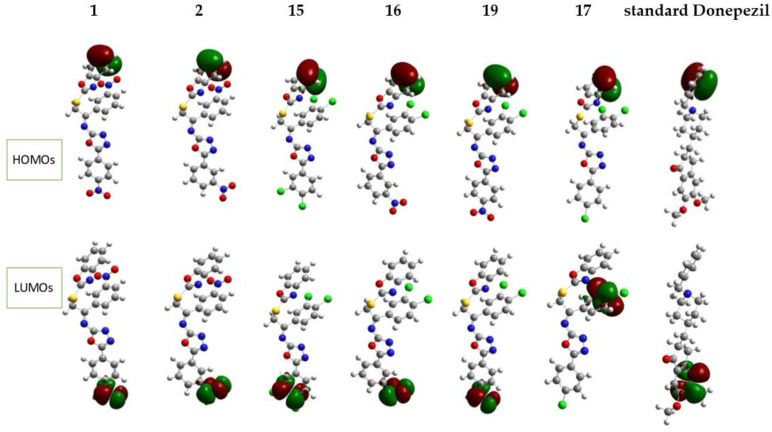
Isodensities of HOMOs and LUMOs of synthesized compounds **1**, **2**, **15**–**17**, **19** and standard drug.

The distribution of the FMOs’ isodensities of all synthesized compounds are shown in Figure 15. The electronic density in HOMOs is distributed mainly on the phenyl group of the benzimidazole ring in all the compounds. However, some electronic density shifts onto the nitro group present next to the benzimidazole ring because of the nitrogen and oxygen so in compounds **1**, **2**, **15**–**17** and **19**, the iso-density also resides on these heteroatoms. As for the standard drug, the density of electrons is mainly on the phenyl group and slightly shifts onto the neighboring nitrogen atom. In all compounds, the other phenyl rings have the greatest distribution of the electronic density in LUMOs (**1**, **2**, **15**, **16** and **19** except **17** and standard drug). These findings show that the electron concentrations are predominantly moving from the donor (the terminal for electron-donating groups) to the recipient unit (the terminal for electron-withdrawing groups). LUMOs are localized near phenyl rings. In the case of compound **17**, the electronic density is migrating to an aromatic ring with substituted chlorines. For standard drugs, electronic density migrates toward the aromatic ring with substituted oxygen atoms. The accepting tendency of the nitro groups causes the HOMO-LUMO band gap for compounds **1** and **19** to be narrower.

#### 2.4.2. Reactivity Descriptor Parameters

Other reactivity descriptor parameters were also examined for additional clarification of the reactivity of the synthesized compounds **1**, **2**, **15**–**17**, **19** and standard drug, and the conclusions of their analysis are displayed in Table 5. These variables include chemical hardness (η), electron affinity (E_A_), ionization potential, electronic chemical potential (µ) and electrophilicity index (ω) [47]. The negative values of HOMOs and LUMOs are equivalent to ionization potential (I) and electron affinity (E_A_), respectively, in accordance with Koopman’s theorem [48].

As evident from FMOs analysis, compounds **1** and **19** show a relation to greater reactivity when related to their chemical hardness. As the η value is little for both these compounds (−1.93 and −1.94 eV, respectively), it shows their enhanced activity towards electrophiles. The electronic affinity values also do not say otherwise that they are the greatest among our synthesized compounds (2.83 and 2.82 eV for **1** and **19**, respectively). Other compounds have smaller electronic affinity with the smallest value of 1.23 eV for standard drug.

The values of both the electronic chemical potential (µ) and electrophilicity index (ω) are highest for compounds **1** and **19** which tell about the transfer of charge on different sites inside the compound. There is extended conjugation inside compounds **1** and **19** due to the presence of aromatic rings leading to instability towards the incoming charges.

#### 2.4.3. Non-Linear Optical (NLO) Properties

Since the dawn of the twenty-first century, researchers have been working on the nonlinear optical (NLO) materials because of their potential uses in lasers, optical data storage, optical communication, optical computing, optical limiting and other technologies. Different strategies are employed to increase the compounds’ NLO responsiveness including the push–pull mechanism, MOFs and EESs [49]. As the value of β_o_ increases due to the transfer of electrons from donor to acceptor groups, the organic molecules exhibit a strong NLO response. This response was studied with the help of polarizability (α_ο_) and hyperpolarizability (β_o_) parameters for the synthesized compounds **1**, **2**, **15**–**17**, **19** and standard drug (Table 6).

Using DFT, these parameters were calculated successfully, and the results are quite comparable with already reported (salicylidenephenyl)benzoxazole compounds [50]. α_ο_ and β_ο_ are highest in the case of compounds **1** and **19** indicating good NLO properties and can be used as an optical material as well as sensory material for optics. As polarizability actually shows the division of electronic charge and density in the chemical system, α_ο_ value is greater for compounds having strong electron donating and withdrawing groups on opposite terminals [51]. Others have moderate values.

As the benzoxazole ring is rich in electrons with the phenolic rings having different attached substituents such as chlorine and nitro groups, the value of β_ο_ is raised by the internal charge transfer.

## 3. Materials and Methods

### 3.1. Materials and Methods

Acetylcholinesterase (E.C.3.1.1.7) (Type VI-S from electric eel and recombinant human enzyme) and butyrylcholinesterase (E.C. 3.1.1.8) from equine serum were purchased from Sigma–Aldrich (Steinheim, Germany). All of the materials used in the synthesis were purchased from Merck or Sigma–Aldrich (St. Louis, MO, USA) (Darmstadt, Germany). The ^1^H NMR and ^13^C NMR spectra in DMSO-*d_6_* were recorded using a Bruker operating at 600 and 150 MHz digital FT-NMR spectrometer (Billerica, MA, USA, Bioscience, Bruker). The NMR spectra’s splitting patterns were recognized and categorized as doublet (d), singlet (s), double doublet (dd), multiplet (m) and triplet (t). In hertz, the coupling constants (J) are given (Hz). Mass spectra were captured on a Shimadzu LCMS-IT-TOF system (Kyoto, Japan) using the electrospray ionization (ESI) method. Using thin-layer chromatography and silica gel 60 F254 to test the purity of synthesized compounds (Darmstadt, Germany, Merck, KGaA).

### 3.2. General Procedure for the Synthesis of Benzoxazole-Based Oxadiazole Analogues (***1***–***19***)

#### 3.2.1. Synthesis of Substituted 2-(Benzo[d]oxazol-2-ylthio)-1-phenylethan-1-one Intermediate (III)

Triethylamine (catalytic amount) was dissolved in ethanol (10 mL). The carbon disulfide (1 mmol) and 2-aminophenol (1 mmol) were added to the solution, and the mixture was allowed to stir at the refluxing temperature. The reaction mixture was refluxed for 5 h and the precipitate was filtered off. The crude product was dried and recrystallized to obtain the 2-marcaptobenzoxazole substrate (II). Then, an intermediate (II) (1 mmol) was treated with different substituted phenacyl bromide (1 mmol) in ethanol (10 mL) and triethylamine (few drops) to afford different substituted 2-(benzo[d]oxazol-2-ylthio)-1-phenylethan-1-one scaffold (III). The crude product was washed, dried and recrystallized from ethanol to give a pure product (III) with an appropriate yield.

#### 3.2.2. Synthesis of Substituted 5-Phenyl-1,3,4-oxadiazol-2-amine Intermediate (VI)

Different substituted benzaldehyde (1 mmol) and semicarbazide (1 mmol) were dissolved in 10 mL of methanol, then the solution was put in a pre-heated sand bath and stirred with a magnetic stirrer. The glacial acetic acid (catalytic amount) was added to this solution. The reaction continued for 8 h. After methanol was removed under reduced pressure, the ice water was added to the residue, and the solid product was filtered off, washed, dried and recrystallized from ethanol to obtain thiosemicarbazone intermediate (V) which further underwent oxidative cyclization with molecular iodine (1.5 mmol) and potassium carbonate (0.75 mmol) in 1,4-dioxane (10 mL). The reaction mixture was kept under reflux for 12 h at refluxing temperature. The completion of the reaction was determined by TLC, and the solvent was evaporated by placing an open container in a fume hood. The resulting residue was washed with water and filtered, dried and recrystallized from ethanol to afford 2-amino-1,3,4-oxadiazole scaffolds (VI).

#### 3.2.3. Synthesis of Targeted Benzoxazole-Based Oxadiazole Scaffolds (**1**–**19**)

Finally, the different substituted 2-(benzo[d]oxazol-2-ylthio)-1-phenylethan-1-one substrate (III) (1 mmol) and 2-amino-1,3,4-oxadiazole intermediates (VI) (1 mmol) were dissolved in acetic acid (10 mL). The reaction mixture was boiled under reflux for 6–8 h, and then subsequently added to ice water and the precipitate so obtained was washed, dried and recrystallized from ethanol to afford targeted benzoxazole-based oxadiazole scaffolds (**1**–**19**) in appropriate yield.

### 3.3. Spectral Analysis

#### 3.3.1. (E)-2-(Benzo[d]oxazol-2-ylthio)-1-(3-nitrophenyl)-N-(5-(4-nitrophenyl)-1,3,4-oxadiazol-2-yl)ethan-1-imine (**1**)

Yield: 63%; brown solid; m.p.: 192–193 °C; ^1^H-NMR (600 MHz, DMSO-*d_6_*): *δ* 8.83 (s, 1H, Ar-H), 8.72 (d, *J* = 8.2 Hz, 2H, Ar-H), 8.65 (dd, *J* = 8.6, 1.3 Hz, 1H, Ar-H), 8.54 (d, *J* = 8.5 Hz, 2H, Ar-H), 8.41 (dd, *J* = 8.0, 1.8 Hz, 1H, Ar-H), 8.06 (t, *J* = 8.7 Hz, 1H, Ar-H), 8.03 (dd, *J* = 7.0, 1.4 Hz, 1H, Benzoxazole-H), 7.99 (dd, *J* = 7.2, 1.6 Hz, 1H, Benzoxazole-H), 7.67 (t, *J* = 7.6, 1.2 Hz, 1H, Benzoxazole-H), 7.64 (t, *J* = 7.6, 1.2 Hz, 1H, Benzoxazole-H), 3.82 (s, 2H, -SCH_2_); ^13^C-NMR (150 MHz, DMSO-*d_6_*): *δ* 179.1, 174.4, 174.2, 170.8, 163.0, 160.9, 157.7, 150.3, 150.2, 147.7, 146.3, 146.0, 144.6, 144.3, 141.5, 141.2, 139.3, 138.7, 136.0, 132.9, 127.8, 115.6, 43.8. HR EI-MS: *m*/*z* calcd for C_23_H_14_N_6_O_6_S [M]^+^ 502.0954; Found: 502.0930.

#### 3.3.2. (E)-2-(Benzo[d]oxazol-2-ylthio)-1-(3-nitrophenyl)-N-(5-(3-nitrophenyl)-1,3,4-oxadiazol-2-yl)ethan-1-imine (**2**)

Yield: 59%; brown solid; m.p.: 188–189 °C; ^1^H-NMR (600 MHz, DMSO-*d_6_*): *δ* 8.82 (s, 1H, Ar-H), 8.72 (s, 1H, Ar-H), 8.75 (dd, *J* = 8.4, 2.3 Hz, 1H, Ar-H), 8.66 (dd, *J* = 8.8, 1.5 Hz, 1H, Ar-H), 8.57 (dd, *J* = 8.3, 2.5 Hz, 1H, Ar-H), 8.42 (dd, *J* = 8.5, 1.9 Hz, 1H, Ar-H), 8.08 (t, *J* = 8.5 Hz, 1H, Ar-H), 8.00 (t, *J* = 7.9 Hz, 1H, Ar-H), 8.05 (dd, *J* = 7.2, 1.8 Hz, 1H, Benzoxazole-H), 7.95 (dd, *J* = 7.3, 1.8 Hz, 1H, Benzoxazole-H), 7.72 (t, *J* = 7.8 Hz, 1H, Benzoxazole-H), 7.69 (t, *J* = 7.6 Hz, 1H, Benzoxazole-H), 3.85 (s, 2H, -SCH_2_); ^13^C-NMR (150 MHz, DMSO-*d_6_*): *δ* 181.5, 175.7, 175.4, 172.8, 167.0, 163.9, 159.7, 156.8, 156.5, 155.7, 154.3, 154.2, 150.6, 150.4, 147.8, 147.4, 142.3, 140.2, 137.5, 135.2, 129.4, 122.6, 44.7. HR EI-MS: *m*/*z* calcd for C_23_H_14_N_6_O_6_S[M]^+^ 502.3250; Found: 502.3211.

#### 3.3.3. (E)-2-(Benzo[d]oxazol-2-ylthio)-N-(5-(2,4-dichlorophenyl)-1,3,4-oxadiazol-2-yl)-1-(3-nitrophenyl)ethan-1-imine (**3**)

Yield: 62%; black solid; m.p.: 197–198 °C; ^1^H-NMR (600 MHz, DMSO-*d_6_*): *δ* 8.79 (s, 1H, Ar-H), 8.68 (dd, *J* = 8.8, 2.0 Hz, 1H, Ar-H), 8.64 (dd, *J* = 8.4, 1.8 Hz, 1H, Ar-H), 8.56 (t, *J* = 8.5 Hz, 1H, Ar-H), 8.23 (s, 1H, Ar-H), 8.17 (d, *J* = 8.0 Hz, 1H, Ar-H), 8.10 (dd, *J* = 7.6, 2.0 Hz, 1H, Benzoxazole-H), 8.05 (dd, *J* = 7.7, 2.8 Hz, 1H, Benzoxazole-H), 8.02 (d, *J* = 8.7 Hz, 1H, Ar-H), 7.92 (t, *J* = 7.4 Hz, 1H, Benzoxazole-H), 7.89 (t, *J* = 7.3 Hz, 1H, Benzoxazole-H), 3.80 (s, 2H, -SCH_2_); ^13^C-NMR (150 MHz, DMSO-*d_6_*): *δ* 182.3, 170.4, 170.0, 168.2, 167.3, 165.6, 159.1, 157.8, 157.3, 156.2, 154.8, 154.6, 152.8, 152.5, 149.8, 149.4, 145.3, 143.2, 139.4, 137.2, 128.4, 126.6, 44.2. HR EI-MS: *m*/*z* calcd for C_23_H_13_N_5_Cl_2_O_4_S [M]^+^ 526.4753; Found: 526.4719.

#### 3.3.4. (E)-2-(Benzo[d]oxazol-2-ylthio)-N-(5-(4-chlorophenyl)-1,3,4-oxadiazol-2-yl)-1-(3-nitrophenyl)ethan-1-imine (**4**)

Yield: 64%; white solid; m.p.: 203–204 °C; ^1^H-NMR (600 MHz, DMSO-*d_6_*): *δ* 8.80 (s, 1H, Ar-H), 8.71 (dd, *J* = 8.6, 1.3 Hz, 1H, Ar-H), 8.66 (dd, *J* = 8.3, 2.8 Hz, 1H, Ar-H), 8.61 (d, *J* = 8.3 Hz, 2H, Ar-H), 8.56 (t, *J* = 8.2 Hz, 1H, Ar-H), 8.54 (d, *J* = 8.5 Hz, 2H, Ar-H), 8.37 (dd, *J* = 7.5, 2.0 Hz, 1H, Benzoxazole-H), 8.15 (dd, *J* = 7.6, 1.9 Hz, 1H, Benzoxazole-H), 7.97 (t, *J* = 7.7, 2.2 Hz, 1H, Benzoxazole-H), 7.94 (t, *J* = 7.9, 1.8 Hz, 1H, Benzoxazole-H), 3.72 (s, 2H, -SCH_2_); ^13^C-NMR (150 MHz, DMSO-*d_6_*): *δ* 186.8, 176.4, 176.0, 172.8, 168.0, 164.9, 159.7, 157.4, 157.1, 152.7, 151.3, 148.5, 143.6, 141.3, 140.8, 140.4, 139.7, 138.4, 136.9, 135.9, 127.4, 118.6, 42.8. HR EI-MS: *m*/*z* calcd for C_23_H_14_ClN_5_O_4_S [M]^+^ 491.1987; Found: 491.1923.

#### 3.3.5. (E)-4-(5-((2-(Benzo[d]oxazol-2-ylthio)-1-(3-nitrophenyl)ethylidene)amino)-1,3,4-oxadiazol-2-yl)-N,N-dimethylaniline (**5**)

Yield: 66%; white solid; m.p.: 201–202 °C; ^1^H-NMR (600 MHz, DMSO-*d_6_*): *δ* 8.77 (s, 1H, Ar-H), 8.68 (dd, *J* = 8.2, 2.3 Hz, 1H, Ar-H), 8.61 (dd, *J* = 8.0, 2.1 Hz, 1H, Ar-H), 8.54 (t, *J* = 8.4 Hz, 1H, Ar-H), 8.47 (d, *J* = 8.0 Hz, 2H, Ar-H), 8.38 (dd, *J* = 7.4, 2.8 Hz, 1H, Benzoxazole-H), 8.26 (dd, *J* = 7.7, 2.0 Hz, 1H, Benzoxazole-H), 8.07 (t, *J* = 7.2 Hz, 1H, Benzoxazole-H), 7.95 (t, *J* = 7.0 Hz, 1H, Benzoxazole-H), 7.34 (d, *J* = 8.0 Hz, 2H, Ar-H), 3.70 (s, 2H, -SCH_2_), 3.10 (s, 6H, -N(CH_3_)_2_); ^13^C-NMR (150 MHz, DMSO-*d_6_*): *δ* 178.3, 176.7, 176.2, 175.9, 170.0, 169.9, 165.1, 162.4, 162.1, 159.4, 157.3, 157.2, 145.6, 145.2, 142.8, 142.4, 140.7, 139.4, 133.9, 131.9, 126.4, 122.8, 43.9, 43.5, 40.2. HR-EI-MS: *m*/*z* calcd for C_25_H_20_N_6_O_4_S [M]^+^ 500.2289; Found: 500.2243.

#### 3.3.6. (E)-2-(Benzo[d]oxazol-2-ylthio)-N-(5-(4-(benzyloxy)phenyl)-1,3,4-oxadiazol-2-yl)-1-(p-tolyl)ethan-1-imine (**6**) (Figures S1 and S2)

Yield: 56%; black solid; m.p.: 191–192 °C; ^1^H-NMR (600 MHz, DMSO-*d_6_*): *δ* 8.18 (d, *J* = 8.2 Hz, 1H, Ar-H), 7.97 (dd, *J* = 7.5, 2.3 Hz, 1H, Benzoxazole-H),7.78 (dd, *J* = 7.8, 2.3 Hz, 1H, Benzoxazole-H), 7.58 (d, *J* = 8.3 Hz, 2H, Ar-H), 7.51 (t, *J* = 8.9 Hz, 1H, Ar-H), 7.43 (d, *J* = 7.4 Hz, 2H, Ar-H), 7.18 (d, *J* = 7.6 Hz, 2H, Ar-H), 7.17 (m, *J* = 8.3 Hz, 3H, Ar-H), 7.16 (t, *J* = 7.8, Hz, 1H, Benzoxazole-H), 7.15 (t, *J* = 7.3 Hz, 1H, Benzoxazole-H), 7.09 (d, *J* = 8.0 Hz, 1H, Ar-H),7.08 (d, *J* = 7.6 Hz, 1H, Ar-H),3.77 (s, 2H, OCH_2_), 3.72 (s, 2H, -SCH_2_), 1.68 (s, 3H, Ar-CH_3_). ^13^C-NMR (150 MHz, DMSO-*d_6_*): *δ* 193.7, 174.0, 168.1, 166.4, 158.5, 155.8, 142.0, 141.8, 135.2, 132.7, 131.0, 130.9, 130.7, 130.5, 129.3, 129.0, 128.8, 127.8, 122.0, 121.9, 121.5, 121.4, 120.2, 118.5, 114.4, 113.9, 109.5, 100.4, 63.0, 39.9, 24.6; HR EI-MS: *m*/*z* calcd for C_31_H_24_N_4_O_3_S [M]^+^ 532.7332; Found: 532.7287.

#### 3.3.7. (E)-2-(Benzo[d]oxazol-2-ylthio)-N-(5-(3-nitrophenyl)-1,3,4-oxadiazol-2-yl)-1-(p-tolyl)ethan-1-imine (**7**)

Yield: 73%; yellow solid; m.p.: 186–188 °C; ^1^H-NMR (600 MHz, DMSO-*d_6_*): *δ* 8.75 (s, 1H, Ar-H), 8.65 (dd, *J* = 8.0, 2.9 Hz, 1H, Ar-H), 8.63 (dd, *J* = 8.1, 2.0 Hz, 1H, Ar-H), 8.55 (t, *J* = 8.0 Hz, 1H, Ar-H), 8.51 (d, *J* = 8.3 Hz, 2H, Ar-H), 8.31 (dd, *J* = 7.5, 1.8 Hz, 1H, Benzoxazole-H), 8.26 (dd, *J* = 7.4, 2.3 Hz, 1H, Benzoxazole-H), 8.09 (t, *J* = 7.3 Hz, 1H, Benzoxazole-H), 8.02 (t, *J* = 7.4 Hz, 1H, Benzoxazole-H), 7.77 (d, *J* = 6.7 Hz, 2H, Ar-H), 3.72 (s, 2H, -SCH_2_), 2.63 (s, 3H, Ar-CH_3_); ^13^C-NMR (150 MHz, DMSO-*d_6_*): *δ* 188.5, 177.7, 177.4, 176.1, 172.0, 170.9, 168.1, 166.3, 165.1, 158.4, 156.3, 157.2, 150.6, 148.2, 147.4, 143.4, 142.7, 142.4, 133.5, 133.2, 129.3, 128.8, 43.9, 24.4. HR EI-MS: *m*/*z* calcd for C_24_H_17_N_5_O_4_S [M]^+^ 471.6381; Found: 471.6323.

#### 3.3.8. (E)-4-(5-((2-(Benzo[d]oxazol-2-ylthio)-1-(p-tolyl)ethylidene)amino)-1,3,4-oxadiazol-2-yl)-N,N-dimethylaniline (**8**) (Figures S3 and S4)

Yield: 60%; white solid; m.p.: 195–196 °C; ^1^H-NMR (600 MHz, DMSO-*d_6_*): *δ* 8.61 (dd, *J* = 7.9, 1.9 Hz, 1H, Benzoxazole-H), 8.26 (d, *J* = 8.0 Hz, 2H, Ar-H), 7.84 (dd, *J* = 7.7, 2.0 Hz, 1H, Benzoxazole-H), 7.70 (t, *J* = 7.6 Hz, 1H, Benzoxazole-H), 7.59 (t, *J* = 7.8 Hz, 1H, Benzoxazole-H), 7.48 (d, *J* = 8.2 Hz, 2H, Ar-H), 7.24–6.92 (m, *J* = 6.7 Hz, 4H, Ar-H), 3.74 (s, 2H, -SCH_2_), 3.17 (s, 6H, N(CH_3_)_2_), 1.50 (s, 3H, Ar-CH_3_); ^13^C-NMR (150 MHz, DMSO-*d_6_*): *δ* 195.4, 188.4, 147.6, 144.8, 144.2, 132.6, 132.2, 132.1, 131.9, 131.8, 131.6, 131.5, 131.4, 131.3, 131.2, 131.1, 129.7, 128.5, 127.2, 123.3, 123.2, 121.1, 45.6, 40.2, 40.0, 20.1; HR EI-MS: *m*/*z* calcd for C_26_H_23_N_5_O_2_S [M]^+^ 469.1398; Found: 469.1321.

#### 3.3.9. (E)-2-(Benzo[d]oxazol-2-ylthio)-N-(5-(2,4-dichlorophenyl)-1,3,4-oxadiazol-2-yl)-1-(p-tolyl)ethan-1-imine (**9**) (Figures S5 and S6)

Yield: 75%; white solid; m.p.: 205–206 °C; ^1^H-NMR (600 MHz, DMSO-*d_6_*): *δ* 8.52 (s, 1H, Ar-H), 7.69 (dd, *J* = 7.1, 2.9 Hz, 1H, Benzoxazole-H), 7.54 (d, *J* = 8.0 Hz, 2H, Ar-H), 7.44 (d, *J* = 6.7 Hz, 2H, Ar-H),7.30 (d, *J* = 6.7 Hz, 2H, Ar-H),7.16 (dd, *J* = 7.4, 2.1 Hz, 1H, Benzoxazole-H), 6.83 (t, *J* = 7.1 Hz, 1H, Benzoxazole-H), 6.11 (t, *J* = 7.6 Hz, 1H, Benzoxazole-H), 3.81 (s, 2H, -SCH_2_), 3.73 (s, 3H, Ar-CH_3_); ^13^C-NMR (150 MHz, DMSO-*d_6_*): *δ* 185.9, 169.0, 168.6, 168.1, 168.0, 150.3, 149.5, 145.6, 143.1, 140.6, 140.1, 132.2, 132.0, 127.8, 127.5, 125.2, 124.0, 123.9, 122.3, 122.1, 119.1, 109.4, 45.6, 21.6; HR EI-MS: *m*/*z* calcd for C_24_H_16_Cl_2_N_4_O_2_S [M]^+^ 495.1579; Found: 495.1542.

#### 3.3.10. (E)-2-(Benzo[d]oxazol-2-ylthio)-N-(5-(4-nitrophenyl)-1,3,4-oxadiazol-2-yl)-1-(p-tolyl)ethan-1-imine (**10**)

Yield: 72%; yellow solid; m.p.: 197–198 °C; ^1^H-NMR (600 MHz, DMSO-*d_6_*): *δ* 8.69 (d, *J* = 8.3 Hz, 2H, Ar-H), 8.57 (d, *J* = 7.9 Hz, 2H, Ar-H), 8.41 (d, *J* = 8.8 Hz, 2H, Ar-H), 8.33 (dd, *J* = 7.4, 1.5 Hz, 1H, Benzoxazole-H), 8.25 (dd, *J* = 7.8, 2.2 Hz, 1H, Benzoxazole-H) 8.17 (t, *J* = 7.4 Hz, 1H, Benzoxazole-H), 8.04 (t, *J* = 7.5 Hz, 1H, Benzoxazole-H), 7.84 (d, *J* = 6.1 Hz, 2H, Ar-H), 3.62 (s, 2H, -SCH_2_), 2.68 (s, 3H, Ar-CH_3_); ^13^C-NMR (150 MHz, DMSO-*d_6_*): *δ* 189.1, 178.6, 178.3, 177.1, 175.2, 173.2, 169.7, 168.6, 165.9, 163.4, 157.3, 153.2, 153.0, 151.0, 149.4, 146.4, 144.6, 143.2, 139.5, 136.2, 129.3, 127.8, 45.8, 24.7. HR EI-MS: *m*/*z* calcd for C_24_H_17_N_5_O_4_S [M]^+^ 471.3642; Found: 471.3603.

#### 3.3.11. (E)-2-(Benzo[d]oxazol-2-ylthio)-N-(5-(4-chlorophenyl)-1,3,4-oxadiazol-2-yl)-1-(p-tolyl)ethan-1-imine (**11**)

Yield: 74%; white solid; m.p.: 193–194 °C; ^1^H-NMR (600 MHz, DMSO-*d_6_*): *δ* 8.65 (d, *J* = 8.0 Hz, 2H, Ar-H), 8.59 (d, *J* = 7.7 Hz, 2H, Ar-H), 8.47 (d, *J* = 8.3 Hz, 2H, Ar-H), 8.35 (dd, *J* = 7.7, 1.8 Hz, 1H, Benzoxazole-H), 8.23 (dd, *J* = 7.4, 2.6 Hz, 1H, Benzoxazole-H), 8.19 (t, *J* = 7.6 Hz, 1H, Benzoxazole-H), 8.08 (t, *J* = 7.3 Hz, 1H, Benzoxazole-H), 7.89 (d, *J* = 6.9 Hz, 2H, Ar-H), 3.68 (s, 2H, -SCH_2_), 2.60 (s, 3H, Ar-CH_3_); ^13^C-NMR (150 MHz, DMSO-*d_6_*): *δ* 188.2, 177.1, 177., 176.6, 174.4, 172.5, 167.4, 165.7, 163.4, 161.2, 158.3, 156.5, 154.2, 153.8, 149.7, 147.1, 145.3, 143.8, 137.2, 135.2, 128.3, 122.5, 42.4, 24.6; HR EI-MS: *m*/*z* calcd for C_24_H_17_ClN_4_O_2_S [M]^+^ 460.3872; Found: 471.3812.

#### 3.3.12. (E)-4-(5-((2-(Benzo[d]oxazol-2-ylthio)-1-(p-tolyl)ethylidene)amino)-1,3,4-oxadiazol-2-yl)benzaldehyde (**12**)

Yield: 64%; brown solid; m.p.: 187–189 °C; ^1^H-NMR (600 MHz, DMSO-*d_6_*): *δ* 9.80 (s, 1H, CHO), 8.63 (d, *J* = 8.2 Hz, 2H, Ar-H), 8.60 (d, *J* = 7.8 Hz, 2H, Ar-H), 8.50 (d, *J* = 8.0 Hz, 2H, Ar-H), 8.39 (dd, *J* = 7.7, 1.8 Hz, 1H, Benzoxazole-H), 8.24 (dd, *J* = 7.5, 2.8 Hz, 1H, Benzoxazole-H), 8.13 (t, *J* = 7.9 Hz, 1H, Benzoxazole-H), 8.06 (t, *J* = 7.4 Hz, 1H, Benzoxazole-H), 7.86 (d, *J* = 7.9 Hz, 2H, Ar-H), 3.69 (s, 2H, -SCH_2_), 2.51 (s, 3H, Ar-CH_3_); ^13^C-NMR (150 MHz, DMSO-*d_6_*): *δ* 190.5, 180.6, 177.4, 177.2, 176.0, 174.1, 172.8, 169.3, 167.2, 163.7, 161.6, 158.8, 157.5, 155.2, 153.8, 149.9, 147.7, 146.3, 144.8, 139.2, 138.2, 126.3, 124.5, 43.4, 24.9; HR EI-MS: *m*/*z* calcd for C_25_H_18_N_4_O_3_S [M]^+^ 460.3872; Found: 471.3812.

#### 3.3.13. (E)-2-(Benzo[d]oxazol-2-ylthio)-N-(5-(4-(benzyloxy)phenyl)-1,3,4-oxadiazol-2-yl)-1-(m-tolyl)ethan-1-imine (**13**)

Yield: 57%; black solid; m.p.: 201–203 °C; ^1^H-NMR (600 MHz, DMSO-*d_6_*): *δ* 8.82 (d, *J* = 8.0 Hz, 2H, Ar-H), 8.77 (s, 1H, Ar-H), 8.58 (dd, *J* = 8.2, 2.5 Hz, 1H, Ar-H), 8.49 (dd, *J* = 7.1, 2.4 Hz, 1H, Benzoxazole-H), 8.42 (dd, *J* = 7.6, 2.6 Hz, 1H, Benzoxazole-H), 8.34 (dd, *J* = 7.0, 2.4 Hz, 1H, Ar-H), 8.23 (t, *J* = 7.0 Hz, 1H, Ar-H), 8.17 (dd, *J* = 8.9, 1.7 Hz, 2H, Ar-H), 8.11 (t, *J* = 7.3, Hz, 1H, Benzoxazole-H), 8.04 (t, *J* = 7.3 Hz, 1H, Benzoxazole-H), 7.96 (d, *J* = 8.1 Hz, 2H, Ar-H), 7.90 (t, *J* = 8.3 Hz, 1H, Ar-H), 7.86 (t, *J* = 8.0 Hz, 1H, Ar-H), 7.76 (m, *J* = 8.3 Hz, 1H, Ar-H), 5.67 (s, 2H, OCH_2_), 3.79 (s, 2H, -SCH_2_), 2.50 (s, 3H, Ar-CH_3_). ^13^C-NMR (150 MHz, DMSO-*d_6_*): *δ* 187.9, 176.8, 176.5, 175.9, 174.1, 171.9, 169.4, 167.2, 167.0, 164.9, 162.4, 161.8, 160.8, 159.9, 158.3, 153.5, 152.8, 150.5, 147.5, 144.2, 143.4, 143.0, 142.4, 140.7, 138.9, 137.9, 128.5, 126.7, 72.9, 45.5, 28.6; HR EI-MS: *m*/*z* calcd for C_31_H_24_N_4_O_3_S [M]^+^ 532.7332; Found: 532.7287.

#### 3.3.14. (E)-2-(Benzo[d]oxazol-2-ylthio)-N-(5-(naphthalen-2-yl)-1,3,4-oxadiazol-2-yl)-1-(p-tolyl)ethan-1-imine (**14**)

Yield: 55%; black solid; m.p.: 205–207 °C; ^1^H-NMR (600 MHz, DMSO-*d_6_*): *δ* 8.56 (s, 1H, Naphthalene-H), 8.51 (dd, *J* = 6.9, 1.6 Hz, Naphthalene-H), 8.47 (dd, *J* = 6.8, 1.8 Hz, Naphthalene-H), 8.43 (d, *J* = 8.2 Hz, 1H, Naphthalene-H), 8.39 (d, *J* = 8.0 Hz, 1H, Naphthalene-H), 8.34 (dd, *J* = 7.1, 2.6 Hz, 1H, Benzoxazole-H), 8.27 (dd, *J* = 7.4, 2.8 Hz, 1H, Benzoxazole-H), 8.19 (t, *J* = 8.2 Hz, 1H, Naphthalene-H), 8.10 (t, *J* = 8.5 Hz, 1H, Naphthalene-H), 8.01 (d, *J* = 8.7 Hz, 2H, Ar-H), 7.97 (d, *J* = 8.4 Hz, 2H, Ar-H), 8.01 (t, *J* = 7.1 Hz, 1H, Benzoxazole-H), 7.98 (t, *J* = 7.6 Hz, 1H, Benzoxazole-H), 3.79 (s, 2H, -SCH_2_), 2.58 (s, 3H, Ar-CH_3_); ^13^C-NMR (150 MHz, DMSO-*d_6_*): *δ* 192.8, 181.4, 181.2, 180.2, 178.2, 175.4, 171.7, 169.8, 167.5, 165.0, 163.9, 161.3, 159.5, 157.6, 150.8, 150.6, 148.7, 148.2, 145.2, 141.8, 138.1, 129.3, 43.8, 21.9. HR EI-MS: *m*/*z* calcd for C_28_H_20_N_4_O_2_S [M]^+^ 476.1739; Found: 476.1702.

#### 3.3.15. (E)-2-(Benzo[d]oxazol-2-ylthio)-1-(3,4-dichlorophenyl)-N-(5-(3,4-dichlorophenyl)-1,3,4-oxadiazol-2-yl)ethan-1-imine (**15**)

Yield: 76%; white solid; m.p.: 198–200 °C; ^1^H-NMR (600 MHz, DMSO-*d_6_*): *δ* 8.46 (s, 1H, Ar-H), 8.41 (s, 1H, Ar-H), 8.40 (d, *J* = 8.2 Hz, 1H, Ar-H), 8.33 (d, *J* = 7.8 Hz, 1H, Ar-H), 8.25 (d, *J* = 7.2 Hz, 1H, Ar-H), 8.18 (dd, *J* = 7.2, 2.0 Hz, 1H, Benzoxazole-H), 8.13 (dd, *J* = 7.0, 2.0 Hz, 1H, Benzoxazole-H), 8.06 (d, *J* = 7.3 Hz, 1H, Ar-H), 7.91 (t, *J* = 7.02 Hz, 1H, Benzoxazole-H), 7.80 (t, *J* = 7.05 Hz, 1H, Benzoxazole-H), 3.77 (s, 2H, -SCH_2_); ^13^C-NMR (150 MHz, DMSO-*d_6_*): *δ* 190.4, 179.4, 179.1, 178.5, 177.2, 174.9, 171.8, 169.2, 166.7, 165.9, 160.3, 150.2, 155.6, 151.2, 149.8, 148.4, 145.7, 144.2, 137.6, 136.6, 124.3, 121.8, 40.8;HR EI-MS: *m*/*z* calcd for C_23_H_12_Cl_4_N_4_O_2_S [M]^+^ 550.1269; Found: 550.1230.

#### 3.3.16. (E)-2-(Benzo[d]oxazol-2-ylthio)-1-(3,4-dichlorophenyl)-N-(5-(3-nitrophenyl)-1,3,4-oxadiazol-2-yl)ethan-1-imine (**16**)

Yield: 68%; white solid; m.p.: 191–193 °C; ^1^H-NMR (600 MHz, DMSO-*d_6_*): *δ* 8.84 (s, 1H, Ar-H), 8.78 (dd, *J* = 7.8, 2.3 Hz, 1H, Ar-H), 8.71 (dd, *J* = 7.9, 1.7 Hz, 1H, Ar-H), 8.63 (t, *J* = 7.5 Hz, 1H, Ar-H), 8.52 (s, 1H, Ar-H), 8.47 (d, *J* = 7.9 Hz, 1H, Ar-H), 8.40 (dd, *J* = 7.8, 2.1 Hz, 1H, Benzoxazole-H), 8.32 (dd, *J* = 7.0, 2.3 Hz, 1H, Benzoxazole-H), 7.92 (d, *J* = 8.7 Hz, 1H, Ar-H), 7.83 (t, *J* = 7.4 Hz, 1H, Benzoxazole-H), 7.74 (t, *J* = 7.0 Hz, 1H, Benzoxazole-H), 3.86 (s, 2H, -SCH_2_); ^13^C-NMR (150 MHz, DMSO-*d_6_*): *δ* 182.8, 170.6, 170.8, 167.9, 167.7, 165.1, 158.1, 157.2, 157.0, 156.9, 154.2, 154.3, 152.4, 152.7, 148.8, 148.4, 144.3, 142.2, 138.4, 137.9, 128.1, 125.6, 44.0. HR EI-MS: *m*/*z* calcd for C_23_H_13_N_5_Cl_2_O_4_S [M]^+^ 526.1759; Found: 526.1703.

#### 3.3.17. (E)-2-(Benzo[d]oxazol-2-ylthio)-N-(5-(4-chlorophenyl)-1,3,4-oxadiazol-2-yl)-1-(3,4-dichlorophenyl)ethan-1-imine (**17**)

Yield: 67%; white solid; m.p.: 183–186 °C; ^1^H-NMR (600 MHz, DMSO-*d_6_*): *δ* 8.81 (s, 1H, Ar-H), 8.78 (d, *J* = 7.9 Hz, 1H, Ar-H), 8.72 (d, *J* = 7.7 Hz, 1H, Ar-H), 8.61 (d, *J* = 7.1 Hz, 2H, Ar-H), 8.47 (dd, *J* = 7.0, 2.3 Hz, 1H, Benzoxazole-H), 8.39 (dd, *J* = 7.9, 2.0 Hz, 1H, Benzoxazole-H), 8.22 (d, *J* = 8.0 Hz, 2H, Ar-H), 8.08 (t, *J* = 7.5 Hz, 1H, Benzoxazole-H), 7.94 (t, *J* = 7.4 Hz, 1H, Benzoxazole-H), 3.62 (s, 2H, -SCH_2_); ^13^C-NMR (150 MHz, DMSO-*d_6_*): *δ* 185.8, 173.6, 173.4, 170.3, 169.7, 169.1, 165.3, 165.2, 162.7, 161.9, 159.2, 153.3, 151.8, 150.7, 148.6, 148.2, 145.3, 144.2, 137.0, 135.9, 129.2, 126.9, 44.7. HR EI-MS: *m*/*z* calcd for C_23_H_13_N_4_Cl_3_O_2_S [M]^+^ 515.3750; Found: 515.3701.

#### 3.3.18. (E)-4-(5-((2-(Benzo[d]oxazol-2-ylthio)-1-(3,4-dichlorophenyl)ethylidene)amino)-1,3,4-oxadiazol-2-yl)-N,N-dimethylaniline (**18**)

Yield: 69%; white solid; m.p.: 193–194 °C; ^1^H-NMR (600 MHz, DMSO-*d_6_*): *δ* 8.87 (s, 1H, Ar-H), 8.81 (d, *J* = 7.3 Hz, 1H, Ar-H), 8.77 (d, *J* = 7.2 Hz, 1H, Ar-H), 8.62 (dd, *J* = 7.9, 2.0 Hz, 1H, Benzoxazole-H), 8.56 (dd, *J* = 7.2, 2.3 Hz, 1H, Benzoxazole-H), 8.51 (d, *J* = 7.1 Hz, 2H, Ar-H), 8.38 (t, *J* = 7.2 Hz, 1H, Benzoxazole-H), 8.30 (t, *J* = 7.5 Hz, 1H, Benzoxazole-H), 7.68 (d, *J* = 8.1 Hz, 2H, Ar-H), 3.57 (s, 2H, -SCH_2_), 3.23 (s, 6H, N(CH_3_)_2_); ^13^C-NMR (150 MHz, DMSO-*d_6_*): *δ* 185.2, 174.6, 174.2, 172.3, 170.6, 170.2, 169.3, 168.2, 167.7, 164.4, 160.2, 158.3, 157.8, 155.2, 151.6, 149.4, 146.3, 143.2, 138.0, 131.2, 129.8, 123.9, 45.4, 45.0, 40.6; HR EI-MS: *m*/*z* calcd for C_25_H_19_N_5_Cl_2_O_2_S [M]^+^ 524.3920; Found: 524.3861.

#### 3.3.19. (E)-2-(Benzo[d]oxazol-2-ylthio)-1-(3,4-dichlorophenyl)-N-(5-(4-nitrophenyl)-1,3,4-oxadiazol-2-yl)ethan-1-imine (**19**)

Yield: 71%; yellow solid; m.p.: 193–194 °C; ^1^H-NMR (600 MHz, DMSO-*d_6_*): *δ* 8.81 (d, *J* = 7.2 Hz, 2H, Ar-H), 8.68 (d, *J* = 7.1 Hz, 2H, Ar-H), 8.87 (s, 1H, Ar-H), 8.82 (d, *J* = 7.8 Hz, 1H, Ar-H), 8.72 (d, *J* = 7.09 Hz, 1H, Ar-H), 8.32 (dd, *J* = 7.7, 2.4 Hz, 1H, Benzoxazole-H), 8.18 (dd, *J* = 7.2, 2.7 Hz, 1H, Benzoxazole-H), 7.98 (t, *J* = 7.7 Hz, 1H, Benzoxazole-H), 7.67 (t, *J* = 7.0 Hz, 1H, Benzoxazole-H), 3.41 (s, 2H, -SCH_2_); ^13^C-NMR (150 MHz, DMSO-*d_6_*): *δ* 188.2, 177.6, 177.1, 175.0, 173.0, 171.9, 169.8, 168.9, 167.2, 164.6, 163.2, 159.3, 158.8, 156.7, 154.5, 149.8, 145.3, 144.3, 134.0, 132.2, 126.8, 113.7, 39.8; HR EI-MS: *m*/*z* calcd for C_23_H_13_N_5_Cl_2_O_4_S [M]^+^ 526.3650; Found: 526.3611.

### 3.4. Assay Protocol for Acetylcholinesterase Inhibition

The inhibition of AChE and BChE was determined using a method described earlier. Briefly, the stock solutions (1 mg/mL) of test analogues were prepared using DMSO. The working solutions (1–100 µg/mL) were prepared using serial dilutions (a serial dilution means a series of diluted solutions, e.g., 0.1 mg/mL, 0.2 mg/mL and so on; the solutions contained 5% DMSO and 95% water). The various concentrations of test compounds (10 µL) were pre-incubated with sodium phosphate buffer (0.1 M; pH 8.0; 150 µL) and AChE (0.1 U/mL; 20 µL) for 15 min at 25 °C. The reaction was initiated via the addition of DTNB (1 mM; 10 µL) and AChEI (1 mM; 10 µL). The mixture of reaction was mixed using a cyclomixer and incubated for 10 min at 25 °C. The absorbance was measured using a microplate reader at a 410 nm wavelength against the blank reading containing 10 µL DMSO instead of the test compound (the solution contained 5% DMSO and 95% water). Utilizing the formula given in Equation (1), the percentage of inhibition was computed and the IC50 was determined under the positive control of Donepezil (0.01–100 g/mL).

% Inhibition = (Absorbance of control − Absorbance of compound)/Absorbance of control × 100.(1)

IC_50_ is the concentration of a drug or inhibitor required to inhibit 50% of an enzyme’s activity which was calculated by constructing a non-linear regression graph between the percentages of inhibition vs. concentration, using Graph Pad prism software (version 5.3) [52,53].

### 3.5. Assay Protocol for Molecular Docking

The MOE software program was used in molecular docking to determine how synthesized analogues interact with both targeted enzymes (AChE and BuChE) to triangulate the outcomes from in vitro and in silico analysis. Using the PDB codes 1ACL for AChE and 1P0P for BuChE, the RCSB protein databank’s crystal structures for both targets were retrieved. The crystallographic structures and all synthesized analogues were protonated using the default MOE-Dock module parameters, resulting in analogue structures and optimized enzymes. After this, a docking study was conducted using the optimized enzyme and analogue structures. Our earlier investigations contain all of the detailed information about the docking process [54,55,56].

## 4. Conclusions

Benzoxazole-based oxadiazole derivatives (**1**–**19**) were synthesized. The novel synthesized compounds were characterized by spectral (^1^H-NMR, ^13^C-NMR and HREI-MS) data and were screened for AChE and BuChE activities. The results are correlated with docking studies. The molecular docking data provided a positive correlation with in vitro AChE and BuChE activities and hence results revealed that these compounds can act as potential inhibitors. The core nucleus benzoxazole-based oxadiazole (**1**–**19**) was efficiently synthesized by treating S-substituted benzoxazole and 2-amino-oxadiazole intermediates. This schiff base formation reaction was preceded very smoothly, in a short reaction time with an excellent yield. Benzoxazole-based oxadiazole derivatives (**1**–**19**) showed good binding interactions with the target enzyme with the least binding energies. The degree of activity and docking studies displayed by the novel innovative structural hybrids of benzoxazole and oxadiazole moieties make these compounds new active leads and promising candidates for the development of anti-Alzheimer agents.

## Figures and Tables

**Table 1:** The types of interactions between receptors and active compounds from varied distance along with docking score.

Active Analogues	Receptor	Types of Interactions	Distance (A^o^)	Docking Score
**Analogue-2 (A)** in AChE complex	TRP-A-58	Pi-S	6.70	−12.83
	TRP-A-58	Pi-Pi Stacked	6.74	
	HIS-A-305	H-B	3.99	
	HIS-A-299	Pi-S	6.68	
	TRP-A-59	Pi-Pi Stacked	5.89	
	TRP-A-59	Pi-Pi Stacked	4.64	
	LEU-A-162	Pi-R	5.65	
	LEU-A-162	Pi-R	5.96	
	GLU-A-233	Pi-S	6.08	
	ALA-A-198	Pi-R	4.52	
	ILE-A-235	Pi-R	6.42	
	ILE-A-235	Pi-R	5.33	
	HIS-A-201	Pi-S	6.31	
	TYR-A-151	Pi-Pi Stacked	6.90	
	GLY-A-306	H-B	3.55	
**Analogue-2 (B)** in BuChE complex	ASP-A-232	H-B	4.28	−12.43
	LYS-A-506	Pi-Anion	4.04	
	LYS-A-506	Pi-R	5.64	
	LYS-A-506	H-B	5.26	
	ILE-A-233	Pi-R	5.22	
	ILE-A-233	C-H	3.69	
	ASN-A-237	H-B	4.82	
	ALA-A-234	H-B	3.52	
	ALA-A-234	Pi-R	5.87	
	TRP-A-329	Pi-Pi T-Shaped	7.71	
	TRP-A-329	Pi-Pi T-Shaped	6.69	
	PHE-A-601	Pi-Pi T-Shaped	7.10	
	ASP-A-568	Pi-Anion	5.79	
	ASP-A-568	Pi-Anion	5.67	
	TRP-A-432	Pi-Sigma	6.88	
	ARG-A-552	H-B	6.25	
**Analogue-15 (C)** in AChE complex	HIS-A-305	Pi-R	3.97	−11.87
	LEU-A-162	Pi-R	5.29	
	LEU-A-162	Pi-R	6.14	
	LEU-A-162	Pi-Sigma	7.29	
	LEU-A-162	Pi-R	7.09	
	HIS-A-101	Pi-S	5.47	
	LYS-A-200	Pi-R	4.16	
	HIS-A-201	Pi-S	5.49	
	ILE-A-235	Pi-Sigma	4.02	
	ALA-A-198	Pi-R	5.61	
	ALA-A-198	Pi-R	5.13	
	ALA-A-198	Pi-R	6.82	
	GLU-A-233	Pi-S	4.60	
	GLN-A-63	H-B	4.58	
	LEU-A-165	Pi-R	5.50	
	TRP-A-59	Pi-Pi Stacked	3.97	
	TRP-A-59	Pi-R	6.04	
	TRP-A-59	Pi-R	5.96	
**Analogue-15 (D)** in BChE complex	ARG-A-552	H-B	6.01	−11.25
	PHE-A-476	Pi-S	6.26	
	ASP-A-568	Pi-Anion	5.57	
	ASP-A-568	Pi-Anion	6.42	
	ASP-A-568	Pi-Anion	6.01	
	TRP-A-432	Pi-R	7.68	
	PHE-A-601	Pi-R	5.63	
	ALA-A-602	Pi-R	4.70	
	ALA-A-628	Pi-R	4.51	
	LEU-A-240	Pi-R	5.85	
	ILE-A-233	Pi-R	4.16	
	ILE-A-233	Pi-R	4.66	
	ALA-A-234	Pi-R	4.32	
	ALA-A-234	H-B	3.28	
	ALA-A-234	Pi-R	5.35	
**Analogue-16 (E)** in AChE complex	LEU-A-162	Pi-R	5.60	−10.96
	LEU-A-162	Pi-R	5.80	
	LEU-A-162	Pi-R	6.22	
	LYS-A-200	Pi-R	5.57	
	HIS-A-201	Pi-Anion	5.55	
	HIS-A-201	Pi-Anion	6.45	
	ILE-A-235	Pi-Sigma	5.17	
	ALA-A-198	Pi-R	4.44	
	ALA-A-198	Pi-R	6.70	
	GLN-A-63	H-B	4.32	
	LEU-A-165	Pi-Sigma	5.37	
	TRP-A-59	Pi-Pi Stacked	4.17	
	TRP-A-59	H-B	4.64	
	HIS-A-305	Pi-R	3.93	
**Analogue-16 (F)** in BuChE complex	PHE-A-601	Pi-Pi Stacked	7.18	−10.16
	TRP-A-329	Pi-Pi Stacked	6.82	
	ASP-A-568	Pi-Anion	5.75	
	ASP-A-568	Pi-Anion	5.71	
	TRP-A-432	Pi-Sigma	6.88	
	ARG-A-552	H-B	6.41	
	ASP-A-232	H-B	3.39	
	LYS-A-506	Pi-Anion	4.44	
	PHE-A-476	Pi-R	4.78	
	LYS-A-506	Pi-Anion	5.26	
	ILE-A-233	Pi-R	6.06	
	PHE-A-476	Pi-R	5.19	
	PHE-A-476	Pi-Pi Stacked	4.28	

**Table 2:** Different substituents (R and R_1_) and in vitro acetylcholinesterase and butyrylcholinesterase inhibitory activities of synthesized benzoxazole-based oxadiazole analogues (**1**–**19**).

Synthesized Compounds	R	R_1_	AChE Inhibition IC_50_ ± SEM ^a^ [µM]	BuChE Inhibition IC_50_ ± SEM ^a^ [µM]
**1**	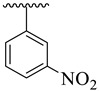	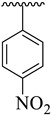	8.60 ± 1.70	8.90 ± 1.80
**2**	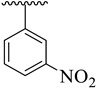	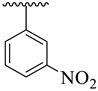	6.40 ± 1.10	7.50 ± 1.20
**3**	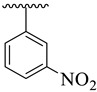	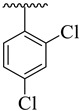	7.80 ± 2.10	9.20 ± 1.90
**4**	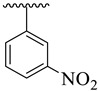	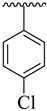	11.20 ± 1.10	11.90 ± 1.10
**5**	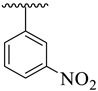	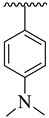	17.70 ± 2.10	16.60 ± 2.50
**6**	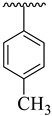	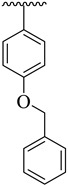	26.30 ± 4.10	28.60 ± 3.80
**7**	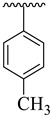	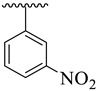	13.60 ± 2.80	15.80 ± 3.10
**8**	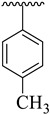	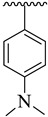	21.70 ± 2.60	24.60 ± 3.80
**9**	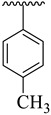	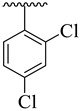	12.30 ± 1.40	14.80 ± 2.70
**10**	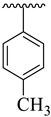	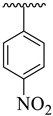	19.40 ± 2.70	18.50 ± 1.90
**11**	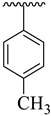	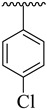	15.80 ± 1.40	12.30 ± 1.50
**12**	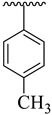	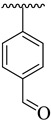	25.70 ± 2.60	28.70 ± 3.90
**13**	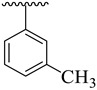	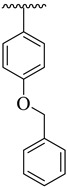	27.90 ± 5.60	31.50 ± 4.90
**14**	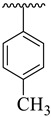	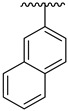	40.80 ± 5.90	42.60 ± 6.10
**15**	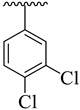	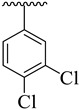	5.80 ± 2.18	7.20 ± 2.30
**16**	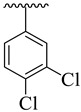	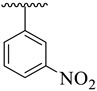	6.90 ± 1.20	7.60 ± 2.10
**17**	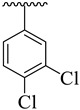	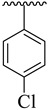	11.40 ± 2.80	13.30 ± 3.20
**18**	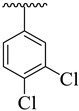	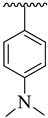	16.40 ± 2.30	18.30 ± 3.20
**19**	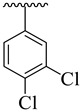	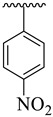	7.20 ± 2.50	9.80 ± 2.30
**Standard Donepezil ^b^ drug**			33.65 ± 3.50	35.80 ± 4.60

Standard error mean (SEM ^a^); Donepezil ^b^ (standard inhibitor for AChE and BuChE activities).

**Table 3:** Optimized structures of synthesized compounds **1**, **2**, **15**–**17**, **19** and standard Donepezil.

**1** 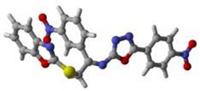	**2** 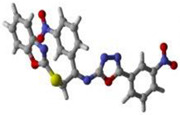	**15** 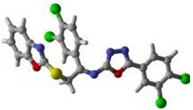	**Standard Donepezil** 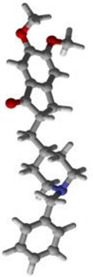
**16** 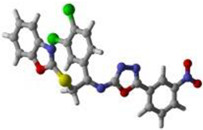	**19** 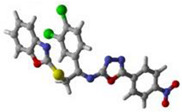	**17** 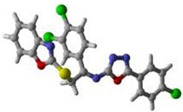	

**Table 4:** Energy of HOMO (E_HOMO_) and LUMO (E_LUMO_), HOMO-LUMO energy gap (Δ_H-L_), dipole moment (μ_ο_) and hyperpolarizability (β_ο_) of compounds.

Compounds	E_HOMO_ (eV)	E_LUMO_ (eV)	Δ_H-L_ (eV)	μ_ο_ (Debye)	β_ο_ (e.s.u.)
**1**	−6.69	−2.83	3.86	4.73	6.91 × 10^−29^
**2**	−6.68	−2.60	4.08	7.62	1.23 × 10^−29^
**15**	−6.69	−2.26	4.42	3.90	1.83 × 10^−29^
**16**	−6.70	−2.59	4.10	10.01	2.23 × 10^−29^
**19**	−6.70	−2.82	3.88	6.81	8.03 × 10^−29^
**17**	−6.65	−2.25	4.40	5.21	2.83 × 10^−29^
**Standard Donepezil**	−5.84	−1.23	4.61	7.61	1.65 × 10^−29^

**Table 5:** Ionization potential (I), electron affinity (E_A_), chemical hardness (η), electronic chemical potential (µ) and electrophilicity index (ω) of synthesized compounds **1**, **2**, **15**–**17**, **19** and standard drug.

Compounds	I (eV)	E_A_ (eV)	η (eV)	µ (eV)	ω (eV)
**1**	6.69	2.83	−1.93	4.76	−5.87
**2**	6.68	2.60	−2.04	4.64	−5.28
**15**	6.69	2.26	−2.21	4.48	−4.53
**16**	6.70	2.59	−2.05	4.64	−5.26
**19**	6.70	2.82	−1.94	4.76	−5.84
**17**	6.65	2.25	−2.20	4.45	−4.50
**Standard Donepezil**	5.84	1.23	−2.30	3.53	−2.71

**Table 6:** Polarizability (α_ο_) and Hyperpolarizability (β_ο_) values of synthesized compounds.

Compounds	α_ο_ (a.u.)	β_ο_ (a.u.)
**1**	538.88	8005.45
**2**	530.58	1429.40
**15**	558.30	2129.64
**16**	542.47	2588.09
**19**	553.44	9302.00
**17**	544.45	3278.25
**Standard Donepezil**	429.28	1919.02

## Data Availability

Data is contained within the article.

## Supplementary Materials

The following supporting information can be downloaded at: https://www.mdpi.com/article/10.3390/ph16070909/s1, Figure S1: ^1^HNMR of compound 6(E)-2-(benzo[d]oxazol-2-ylthio)-N-(5-(4-(benzyloxy)phenyl)-1,3,4-oxadiazol-2-yl)-1-(p-tolyl)ethan-1-imine (**6**); Figure S2: ^13^CNMR of compound 6(E)-2-(benzo[d]oxazol-2-ylthio)-N-(5-(4-(benzyloxy)phenyl)-1,3,4-oxadiazol-2-yl)-1-(p-tolyl)ethan-1-imine (**6**); Figure S3: 1HNMR of compound 8 (E)-4-(5-((2-(benzo[d]oxazol-2-ylthio)-1-(p-tolyl)ethylidene)amino)-1,3,4-oxadiazol-2-yl)-N,N-dimethylaniline (**8**); Figure S4: 13CNMR of compound 8 (E)-4-(5-((2-(benzo[d]oxazol-2-ylthio)-1-(p-tolyl)ethylidene)amino)-1,3,4-oxadiazol-2-yl)-N,N-dimethylaniline (**8**); Figure S5: 1HNMR of compound 9 (E)-2-(benzo[d]oxazol-2-ylthio)-N-(5-(2,4-dichlorophenyl)-1,3,4-oxadiazol-2-yl)-1-(p-tolyl)ethan-1-imine (**9**); Figure S6: 13CNMR of compound 9 (E)-2-(benzo[d]oxazol-2-ylthio)-N-(5-(2,4-dichlorophenyl)-1,3,4-oxadiazol-2-yl)-1-(p-tolyl)ethan-1-imine (**9**).
