# Supplementary material for: Investigation of Novel Benzoxazole-Oxadiazole Derivatives as Effective Anti-Alzheimer’s Agents: In Vitro and In Silico Approaches
Source: Pharmaceuticals (Basel). 2023 Jun 21;16(7):909. doi: 10.3390/ph16070909 (PMC10384982; doi:10.3390/ph16070909)
Supplement: Supplementary file 1 [file pharmaceuticals-16-00909-s001.zip › pharmaceuticals-2432595-supplementary.pdf]

Investigation of Novel Benzoxazole-Oxadiazole Derivatives as Effective Anti-Alzheimer's Agents: *In Vitro* and *In Silico* Approaches

Saeed Anwar[a], Wajid Rehman*[a], Rafaqat Hussain*[a], Shoaib Khan[b], Mohammed M. Alanazi[c], Nawaf A. Alsaif[c], Yousaf Khan[d], Shahid Iqbal[e], Muhammad Ali Hashmi[f] and AdeelaNaz[f]

[a]Department of Chemistry, Hazara University Mansehra-21120, Pakistan.
[b]Department of Chemistry, Abbottabad University of Science and Technology (AUST) Abbottabad, Pakistan.
[C]Department of Pharmaceutical Chemistry, College of Pharmacy, King Saud University, Riyadh 11451, Saudi Arabia
[d]Department of Chemistry, COMSATS University Islamabad, Islamabad 45550, Pakistan.
[e]School of Chemical and Environmental Engineering, College of Chemistry, Chemical Engineering and Materials Science, Soochow University, Suzhou, Jiangsu, 215123, China
[f]Department of Chemistry, Division of Science & Technology, University of Education, 54770 Lahore, Pakistan

Supplimentary Information

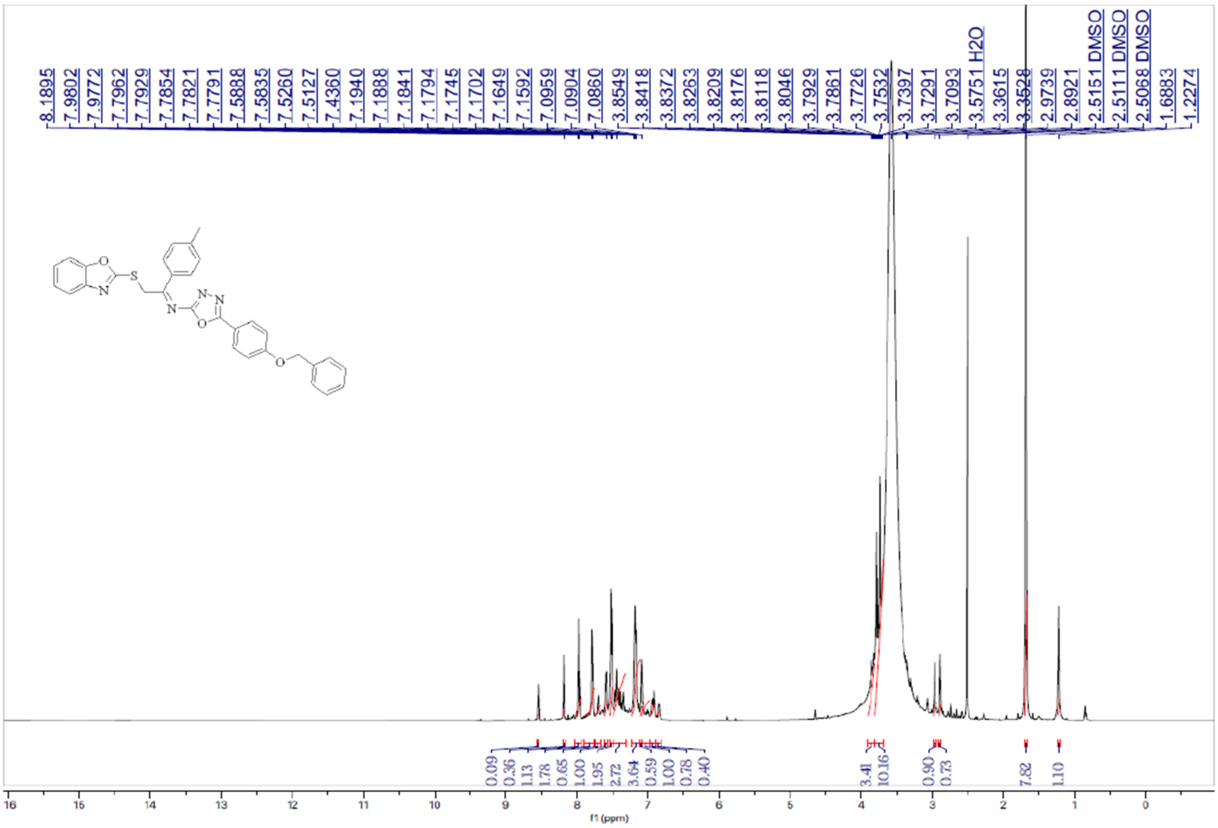

Figure S1.^{1}HNMR of compound 6(*E*)-2-(benzo[d]oxazol-2-ylthio)-N-(5-(4-(benzyloxy)phenyl)-1,3,4-oxadiazol-2-yl)-1-(p-tolyl)ethan-1-imine (**6**)

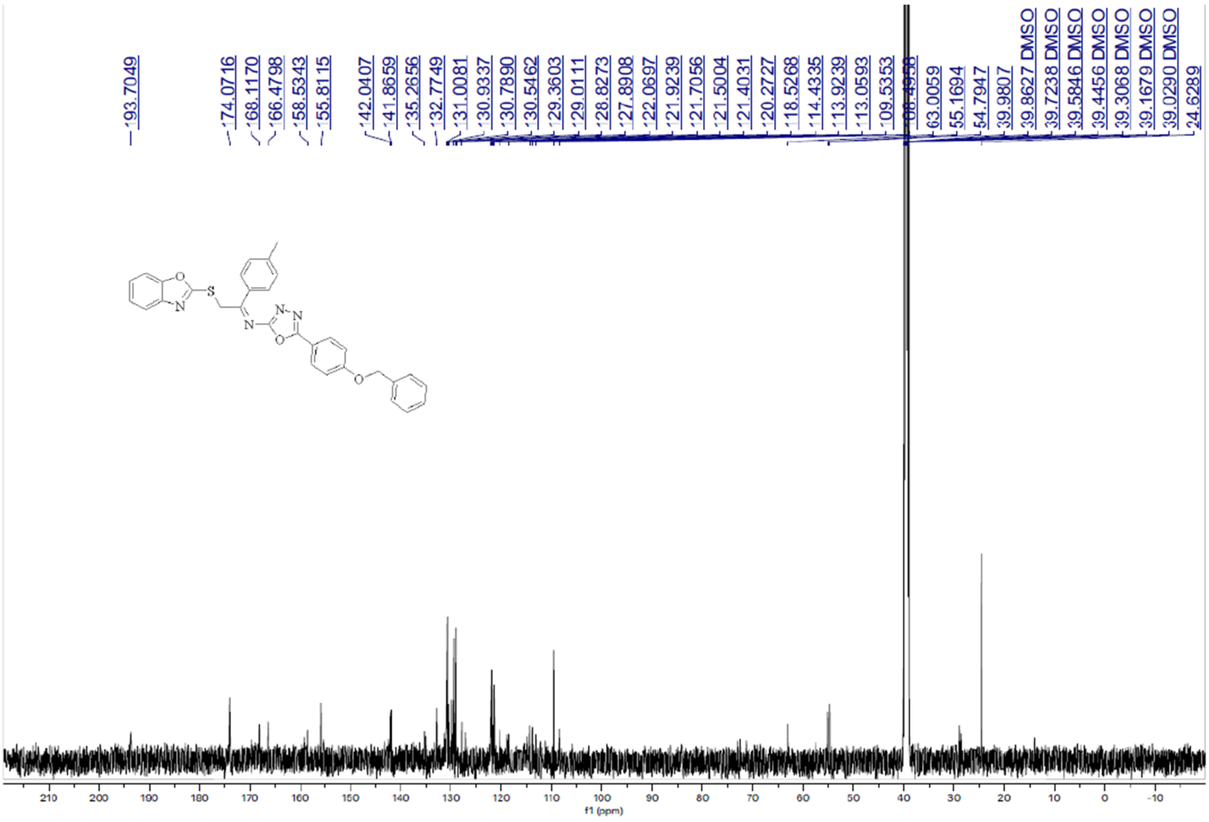

Figure S2.^{13}CNMR of compound 6(*E*)-2-(benzo[d]oxazol-2-ylthio)-N-(5-(4-(benzyloxy)phenyl)-1,3,4-oxadiazol-2-yl)-1-(p-tolyl)ethan-1-imine (6)

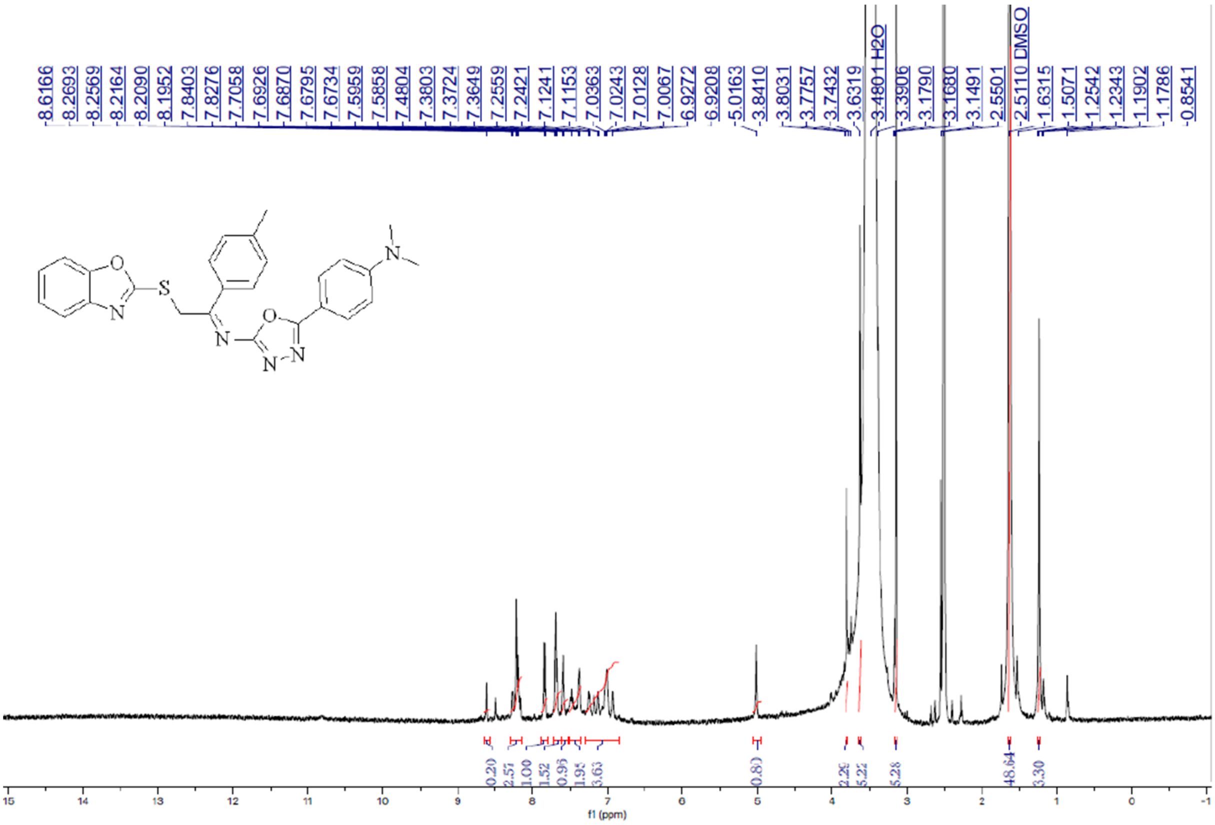

Figure S3.^{1}HNMR of compound 8 (*E*)-4-(5-((2-(benzo[d]oxazol-2-ylthio)-1-(p-tolyl)ethylidene)amino)-1,3,4-oxadiazol-2-yl)-N,N-dimethylaniline (**8**)

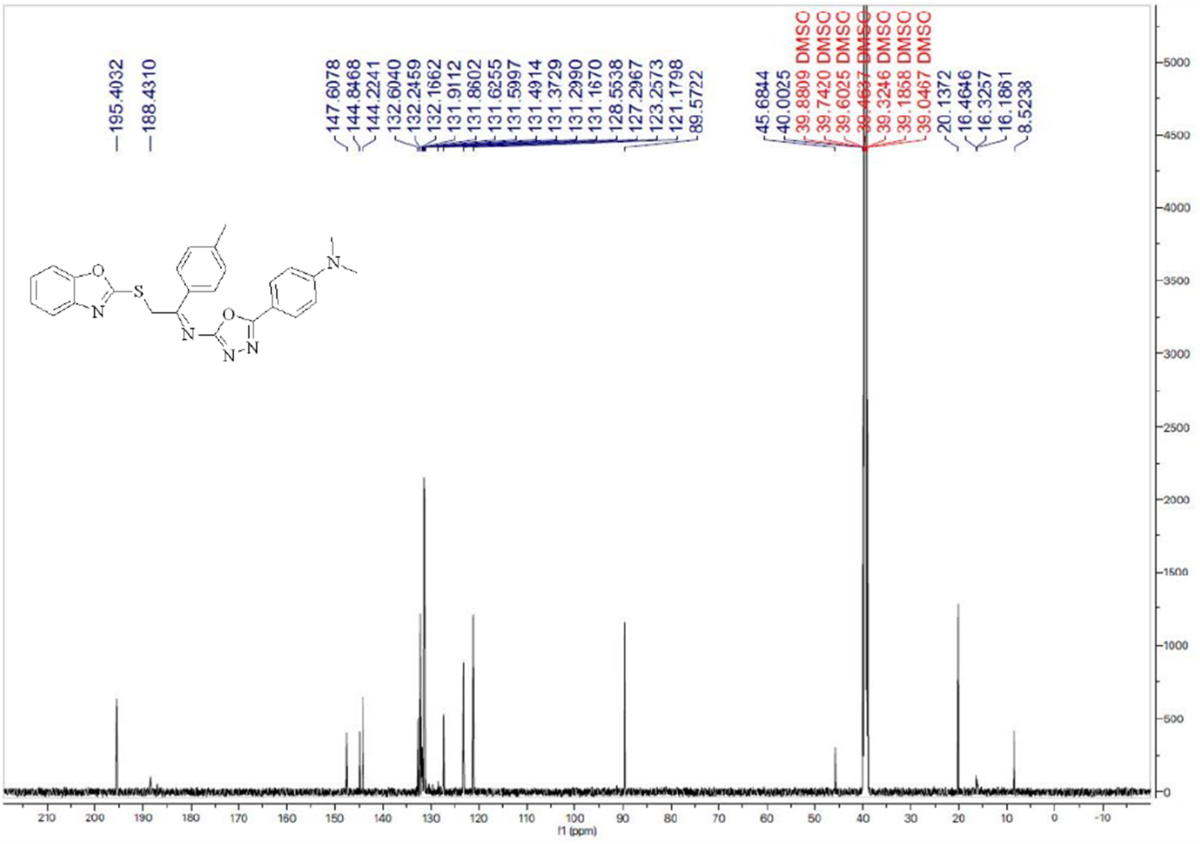

Figure S4.^{13}CNMR of compound 8 (*E*)-4-(5-((2-(benzo[d]oxazol-2-ylthio)-1-(p-tolyl)ethylidene)amino)-1,3,4-oxadiazol-2-yl)-N,N-dimethylaniline (**8**)

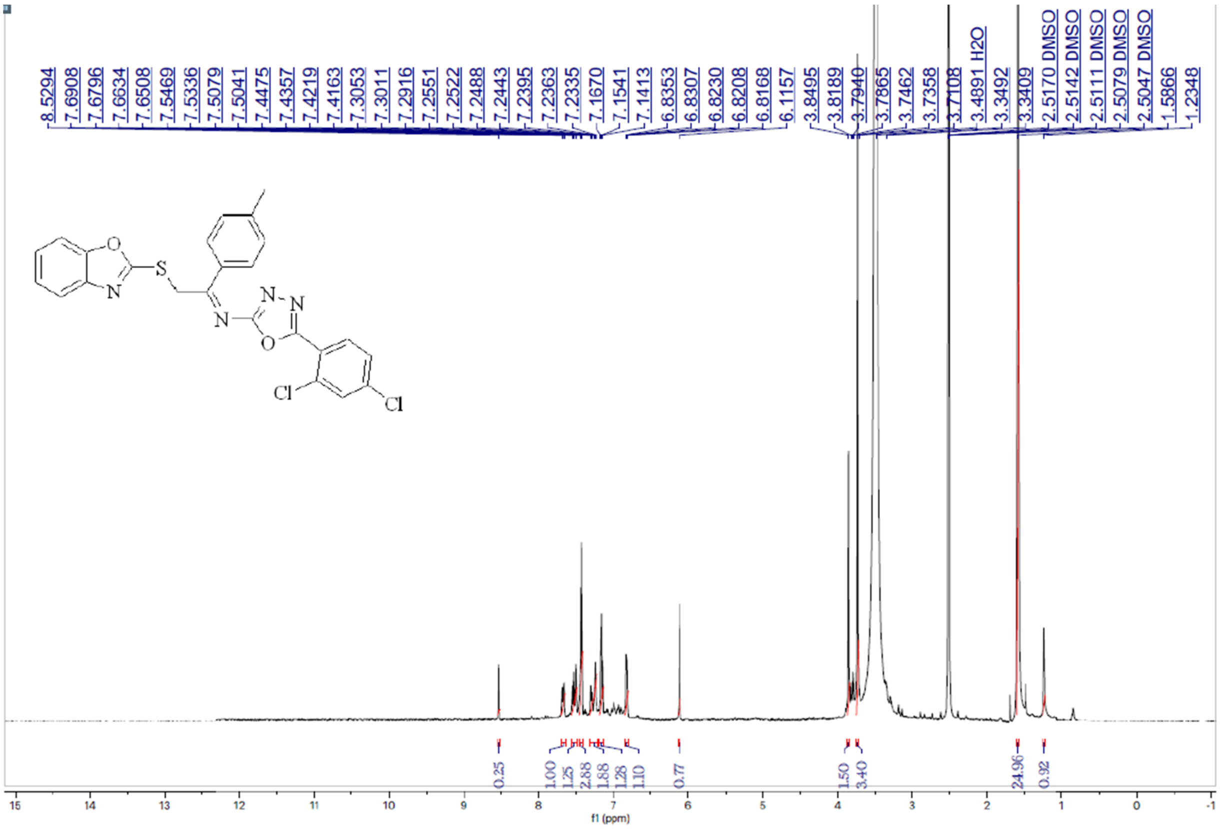

Figure S5.^{1}HNMR of compound 9 (*E*)-2-(benzo[d]oxazol-2-ylthio)-N-(5-(2,4-dichlorophenyl)-1,3,4-oxadiazol-2-yl)-1-(p-tolyl)ethan-1-imine (**9**)

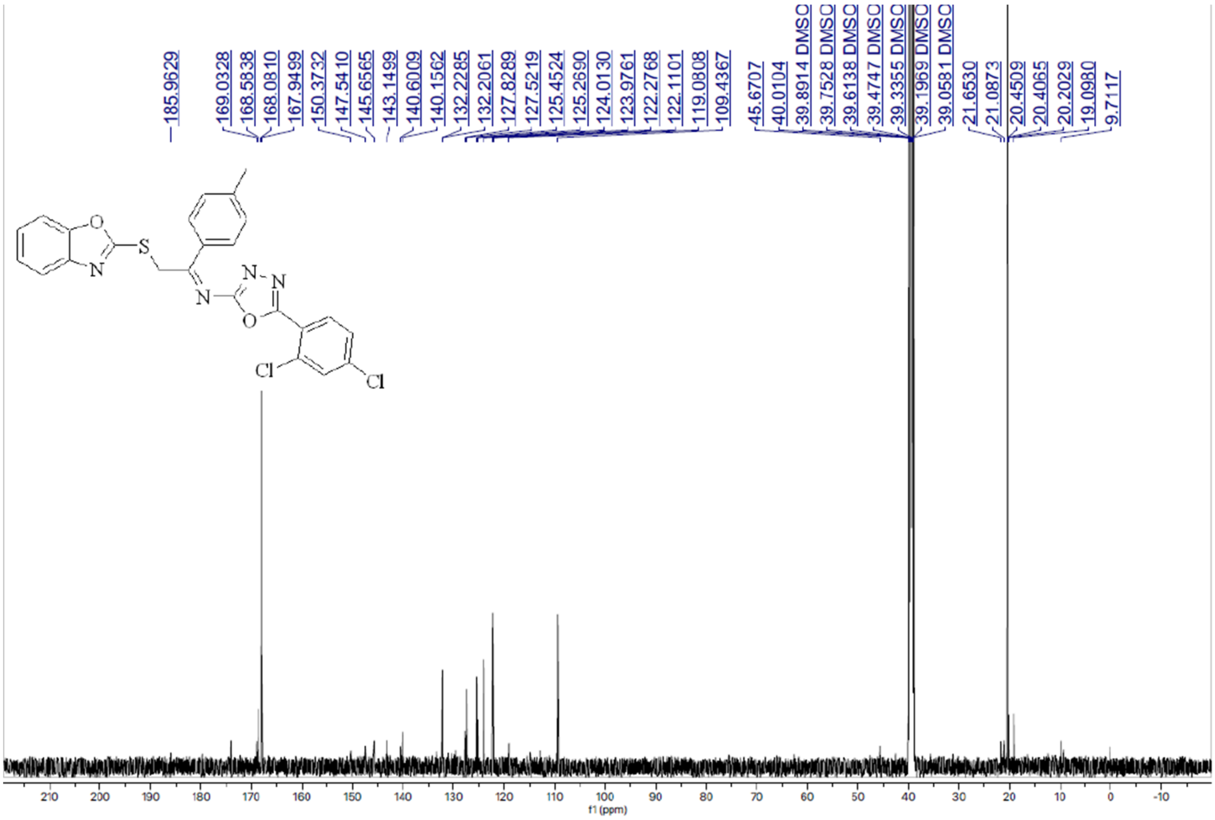

Figure S6. ^{13}CNMR of compound 9 (*E*)-2-(benzo[d]oxazol-2-ylthio)-N-(5-(2,4-dichlorophenyl)-1,3,4-oxadiazol-2-yl)-1-(p-tolyl)ethan-1-imine (**9**)
